# Synthesis and molecular docking of new *N*4-piperazinyl ciprofloxacin hybrids as antimicrobial DNA gyrase inhibitors

**DOI:** 10.1007/s11030-022-10528-z

**Published:** 2022-09-24

**Authors:** Hamada H. H. Mohammed, Doaa Mohamed Elroby Ali, Mohamed Badr, Ahmed G. K. Habib, Abobakr Mohamed Mahmoud, Sarah M. Farhan, Shimaa Salah Hassan Abd El Gany, Soad A. Mohamad, Alaa M. Hayallah, Samar H. Abbas, Gamal El-Din A. Abuo-Rahma

**Affiliations:** 1 Department of Pharmaceutical Chemistry, Faculty of Pharmacy, Sohag University, Sohag, 82524 Egypt; 2 Department of Medicinal Chemistry, Faculty of Pharmacy, Minia University, Minia, 61519 Egypt; 3 Department of Pharmaceutical Chemistry, Faculty of Pharmacy, Deraya University, New Minia City, 61768 Egypt; 4 Department of Biochemistry, Faculty of Pharmacy, Sohag University, Sohag, 82524 Egypt; 5 Department of Biochemistry, Faculty of Pharmacy, Menoufia University, Menoufia, Egypt; 6 Department of Biotechnology and Life Sciences, Faculty of Postgraduate Studies for Advanced Sciences, Beni-Suef University, Beni-Suef, Egypt; 7 Department of Microbiology and Immunology, Faculty of Pharmacy, Deraya University, New Minia City, 61768 Egypt; 8 Department of Pharmaceutics and Clinical Pharmacy, Faculty of Pharmacy, Deraya University, New Minia, Minya, 61768 Egypt; 9 Pharmaceutical Organic Chemistry Department, Faculty of Pharmacy, Assiut University, El Fateh, 71526 Egypt; 10 Pharmaceutical Chemistry Department, Faculty of Pharmacy, Sphinx University, New Assiut, Egypt

**Keywords:** Ciprofloxacin chalcone, Ciprofloxacin pyrimidine, DNA gyrase inhibitors, Antimicrobial

## Abstract

**Abstract:**

A series of *N*-4 piperazinyl ciprofloxacin derivatives as urea-tethered ciprofloxacin-chalcone hybrids **2a-j** and thioacetyl-linked ciprofloxacin-pyrimidine hybrids **5a-i** were synthesized. The target compounds were investigated for their antibacterial activity against *S. aureus*, *P. aeruginosa, E. coli,* and *C. albicans* strains, respectively. Ciprofloxacin derivatives **2a-j** and **5a-i** revealed broad antibacterial activity against either Gram positive or Gram negative strains, with MIC range of 0.06–42.23 µg/mL compared to ciprofloxacin with an MIC range of 0.15–3.25 µg/mL. Among the tested compounds, hybrids **2b**, **2c**, **5a, 5b**, **5h,** and **5i** exhibited remarkable antibacterial activity with MIC range of 0.06–1.53 µg/mL against the tested bacterial strains. On the other hand, compounds **2c**, **2e**, **5c,** and **5e** showed comparable antifungal activity to ketoconazole against *candida albicans* with MIC range of 2.03–3.89 µg/mL and 2.6 µg/mL, respectively. Further investigations showed that some ciprofloxacin hybrids have inhibitory activity against DNA gyrase as potential molecular target compared to ciprofloxacin with IC_50_ range of 0.231 ± 0.01–7.592 ± 0.40 µM and 0.323 ± 0.02 µM, respectively. Docking studies of compounds **2b, 2c, 5b, 5c, 5e, 5h, and 5i** on the active site of DNA gyrase (PDB: 2XCT) confirmed their ability to form stable complex with the target enzyme like that of ciprofloxacin.

**Graphical abstract:**

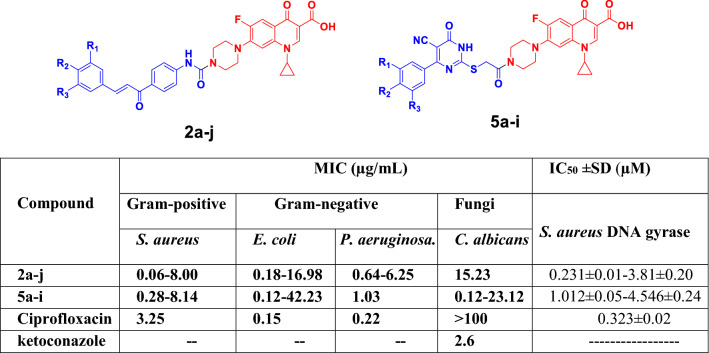

**Supplementary Information:**

The online version contains supplementary material available at 10.1007/s11030-022-10528-z.

## Introduction

**Fig. 1:**
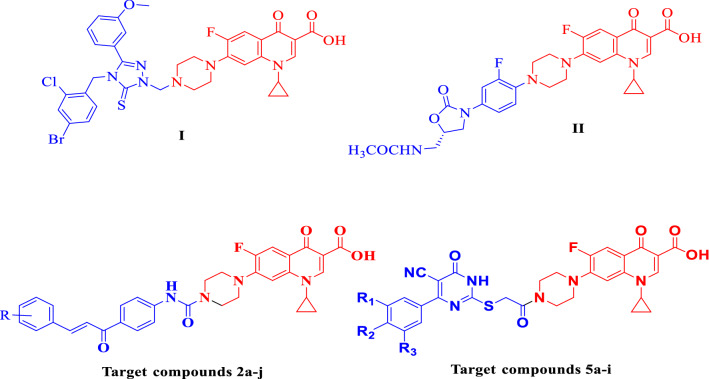
Structures of some reported ciprofloxacin derivatives and target compounds **2a-j** and **5a-i**

The prevalence of bacterial resistance to therapeutically used antibiotics is a global health problem that encouraged scientists to develop new antibiotics [1, 2]. Therefore, novel antibacterial agents that have little or no bacterial resistance are urgently required to improve the efficiency of antibiotics against microbes [3]. One of the important strategies to do this is modifying the structural features of the existing antibiotics [4, 5]. In this situation, the development of antibiotic hybrids can function properly because it requires no validation of novel biological targets or the discovery of new antibacterial pharmacophores [6]. This strategy involves linking two or more antibiotics that inhibit different targets in bacteria into one single molecule [7, 8]. It is believed that hybrid molecules act by inhibiting two or multi targets simultaneously, increasing potency against drug-resistant bacterial strains, expanding the spectrum of activity, and decreasing the possibility of developing bacterial resistance [6, 9]. Ciprofloxacin is a broad-spectrum antibacterial agent with an excellent safety profile [10]. It is widely used for the treatment of bacterial infection, and as a second-line agent for management of tuberculosis (TB) [11]. It achieves its antibacterial effect by inhibiting bacterial DNA gyrase(topoisomerase II) which involved in DNA replication [10, 12]. It was reported that position 7 in quinolones and fluoroquinolones is responsible for interaction with DNA gyrase, or topoisomerase IV [13–15]. The nature of the substituent at position 7 greatly influences spectrum, potency, and pharmacokinetics [16, 17]. The introduction of a bulky substituent at position 7 was found to diminish the likelihood of bacterial resistance in wild-type bacterial strains and improved potency against anaerobic bacteria [9–11]. Recent research studies revealed that substituting the *N*-4 piperazine heterocycle of ciprofloxacin with a lipophilic substituent was found to enhance its antibacterial and antimycobacterial activities [16, 17]. Also, It was reported that 1,2,4-triazole-5(*4H*)-thione ciprofloxacin hybrid **I** revealed remarkable in vitro antibacterial activity against all tested strains, either Gram-positive or Gram-negative pathogens with MIC < 0.24 µg/mL and 0.01 to 1.32 µg/mL, respectively, compared to ampicillin (MIC: 3.9–250 µg/mL) (Fig. 1) [18, 19]. The oxazolidinone-ciprofloxacin hybrid **II** displayed potent in vitro activity against Gram negative and Gram positive with MIC range 0.125–4 µg/mL and remarkable potency against fluoroquinolones, vancomycin, and/or linezolid-resistant strains with MIC range 0.25–0.5 µg/mL (Fig. 1) [20]. On the other hand, Chalcones, known as α,β-unsaturated ketones, are essential building pharmacophores in many drug design protocols [21]. Chalcone-containing compounds are of special interest because of the ease of their synthesis, improved lipophilic profile, and pronounced antimicrobial activities [21–23]. They showed broad spectrum of pharmacological activities such as antibacterial [24], antimalarial [25], antifungal [26], antiviral [27], anti-inflammatory [28], and anticancer activities [29, 30]. Another moiety of high importance in medicinal chemistry is the pyrimidine core [31]. Pyrimidine-containing compounds have been well recognized for their versatile therapeutic applications such as anticancer [32], antimalarial [33], antiviral [34], antibacterial [35, 36], antifungal [37, 38], and anticonvulsant [39]. Based on molecular hybridization, linking of ciprofloxacin at its *N*-4 piperazine moiety with different biologically active scaffolds could result in hybrid molecules that could modulate more than one target, simultaneously enhancing its antibacterial activity.

Considering these issues, and due to the global health concern of bacterial resistance, this work aims to synthesize new ciprofloxacin-chalcone hybrids **2a-j** and new ciprofloxacin-pyrimidine hybrids **5a-j** and evaluation of their antibacterial and antifungal activities. Also, DNA gyrase assay was done as a potential target for the newly synthesized ciprofloxacin hybrids. Moreover, molecular docking studies were carried out to explore their binding modes.

## Results and discussion

### Chemistry

**Scheme 1:**
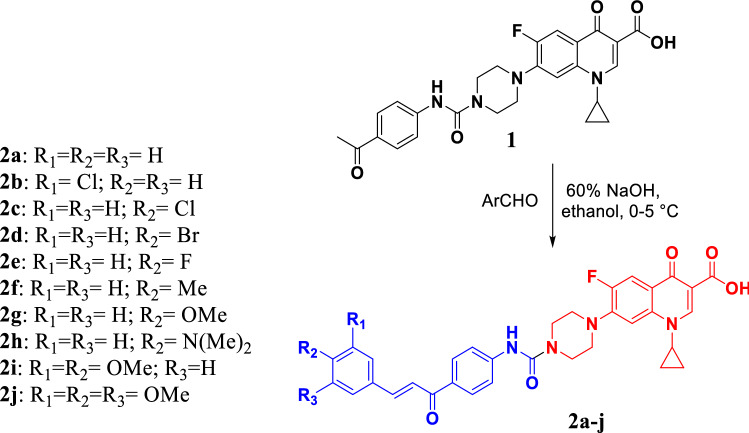
Synthesis of target ciprofloxacin hybrids 2a–j

The *N*-4-piperazinyl ciprofloxacin-chalcones **2a-j** were prepared as reported by Condensation of compound **1** with various aromatic aldehydes in ethanol using 60% solution of sodium hydroxide to afford the target hybrids 2a-j in a good yield, Scheme 1 [30].

Reagents and conditions: (i) Appropriate aromatic aldehydes, 60% NaOH, ethanol, 0–5 °C stirring overnight, then neutralized with dil. acetic acid.

**Scheme 2:**
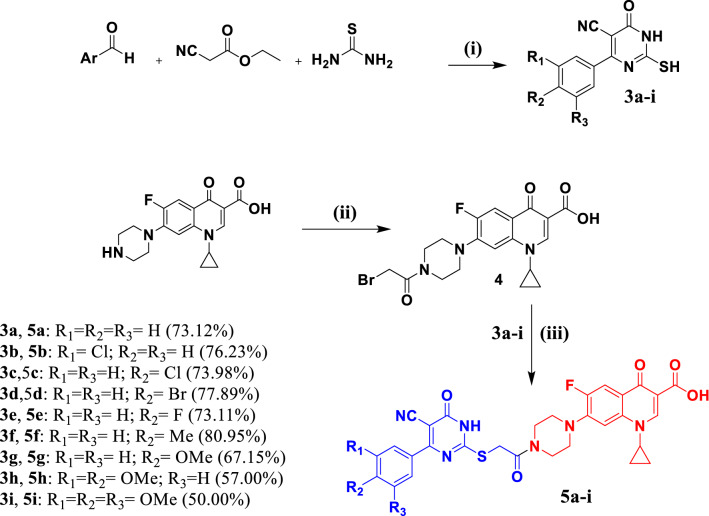
Synthesis of target compounds 5a–i

Moreover, the newly prepared ciprofloxacin-pyrimidine 5a-i were prepared as outlined in Scheme 2.

Reagent s and conditions: (i) Potassium carbonate, ethanol, reflux 8 h; (ii) Bromoacetyl bromide, triethylamine (TEA), dichloromethane, 0–5° C stirring overnight; (iii) pyrimidine intermediate 5a-i, acetonitrile, triethylamine, reflux 4–6 h.

The desired intermediate pyrimidine derivatives **3a-i** were prepared as reported by heating at reflux a mixture of ethyl cyanoacetate, thiourea, and the appropriate aromatic aldehyde in absolute ethanol in the presence of potassium carbonate as a base [40, 41]. Ciprofloxacin derivative **4** was prepared by treating ciprofloxacin with bromoacetyl bromide in dichloromethane at 0 °C in the presence of TEA [42]. Alkylation of pyrimidine derivatives **3a-i** with compound **4** were achieved in acetonitrile in the presence of TEA according to a reported procedure [43, 44] to afford the target new compounds **5a-i**. The ^1^H NMR spectra of compounds **5a-i** displayed the characteristic known pattern of fluoroquinolone scaffold. In addition, two doublets at δ = 7.53–8.67 ppm indicated the presence of *para* disubstituted phenyl ring in their structures as in pyrimidine derivative **5f**. Moreover, compound **5e** showed triplet at δ = 7.38 ppm of (2*H*) Ar–*H* due to fluorine coupling. Meanwhile, compound **5f** displayed singlet at δ = 2.30 ppm corresponding Ar-C*H*_3_. Likewise, the pyrimidine derivative **5 g** revealed a singlet at δ = 3.84 ppm belonging to the *p*-OC*H*_3_ group. Also, **5h** displayed two singlets corresponding the presence of to the two methoxy groups at δ = 3.77 and 3.84 ppm groups. Furthermore, **5i** showed two singlets for the three methoxy groups at δ = 3.78 and 3.85 ppm and a singlet at δ = 7.36 ppm which indicates the presence of 3,4,5-trimethoxyphenyl moiety. ^13^C NMR spectra of compounds **5a-i** revealed the characteristic pattern for fluoroquinolone nucleus and the carbonyl carbon of the thioacetyl linker, carbonyl of the carboxylic group, and C-6 of the dihydropyridine moiety, respectively. Also, ^13^C NMR spectra of compounds **5a-i** showed a characteristic signal at δ = 91.69–93.06 ppm corresponding to the carbonitrile group. Data from the elemental analysis further confirmed the structures of the target compounds. Finally, Mass data for compounds **5a-i** confirmed their assigned structures. The m/z value of molecular ion peak [M-H]^−^ for each compound was close to the calculated.

### Biological Investigation

#### Screening of antibacterial and antifungal activities

The antibacterial and antifungal activities of compounds, ketone **1**, **2a-j,** and **5a-i** were evaluated in vitro against *S. aureus* (ATCC 6538*)*, *P. aeruginosa* (ATCC 10,145)*, E. coli* (ATCC 8739), and *C. albicans* (ATCC 10,231). Hybrids **2a-j** and the newly synthesized ciprofloxacin hybrids 5a-i were evaluated against ciprofloxacin and ketoconazole as antibacterial and antifungal references, respectively using standard agar cup diffusion method [45]. Screening results are listed in Table 1. According to the results recorded in Table 1, it was found that hybrid **1** has a potent antibacterial activity against *S. aureus* compared to ciprofloxacin with MICs of 0.7 µg/mL and 3.25 µg/mL, respectively. Also, it showed considerable activity against both of *E. coli* and *P. aeruginosa* compared to ciprofloxacin with MICs of 0.60 µg/mL, 1.20 µg/mL, 0.15 µg/mL, and 0.22 µg/mL**,** respectively. Regarding the antibacterial activities of ciprofloxacin hybrids **2a-j,** compounds **2a, 2b, 2c, 2e, 2f,** and **2j** exhibited potent antibacterial activities against *S. aureus* compared to ciprofloxacin with MICs of 0.74 µg/mL, 0.06 µg/mL, 0.67 µg/mL, 0.89 µg/mL, 0.94 µg/mL, 1.48 µg/mL, and 3.25 µg/mL, respectively, while hybrids **2a**, **2b**, **2c**, and **2e** showed remarkable antibacterial activities against *E. coli* compared to ciprofloxacin with MICs of 0.64 µg/mL, 0.18 µg/mL, 0.34 µg/mL, 0.53 µg/mL, and 0.15 µg/mL, respectively. Moreover, hybrids **2a**, **2b**, **2c** and **2e** showed moderate activity against *P. aeruginosa* compared to ciprofloxacin with MICs of 1.12 µg/mL, 0.64 µg/mL, 1.03 µg/mL, 0.96 µg/mL, and 0.22 µg/mL**,** respectively.

**Table 1:** In vitro antibacterial and antifungal activities of ciprofloxacin derivatives **2a-j, 5a-i**, ciprofloxacin and ketoconazole

Compound	MIC (µg/mL)			
	Gram-positive	Gram-negative		Fungi
	*S. aureus*	*E. coli*	*P. aeruginosa*	*C. albicans*
**1**	0.70	0.60	1.20	˃100
**2a**	0.74	0.64	1.12	˃100
**2b**	0.06	0.18	0.64	15.23
**2c**	0.67	0.34	1.03	3.89
**2d**	2.65	4.78	7.25	˃100
**2e**	0.89	0.53	0.96	2.03
**2f**	0.94	9.33	3.7	˃100
**2g**	8	16.98	6.25	˃100
**2h**	1.2	5.25	3.125	84.21
**2i**	6.3	4.13	17	. ˃100
**2j**	1.48	5.13	4.2	˃100
**5a**	0.36	0.91	1.53	6.23
**5b**	0.7	0.12	0.19	˃100
**5c**	0.58	1.03	2.04	2.87
**5d**	8.14	42.23	12.30	36.12
**5e**	0.78	0.52	3.25	2.51
**5f**	4.79	3.89	2.95	˃100
**5g**	0.28	14.25	23.12	˃100
**5h**	0.34	0.21	0.49	28.73
**5i**	0.74	0.63	0.13	˃100
**Ciprofloxacin**	3.25	0.15	0.22	˃100
**Ketoconazole**	–	–	–	2.6

Concerning the antibacterial activities of hybrids **5a-i**, ciprofloxacin derivatives **5a**, **5b**, **5c**, **5e**, **5 g**, **5h,** and **5i** exhibited potent antibacterial activities against *S. aureus* compared to ciprofloxacin with MICs of 0.36 µg/mL, 0.7 µg/mL, 0.58 µg/mL, 0.78 µg/mL, 0.28 µg/mL, 0.34 µg/mL, 0.74 µg/mL, and 3.25 µg/mL, respectively. Furthermore, hybrids **5a**, **5b**, **5e**, **5h**, and **5i** revealed moderate activities against *E. coli* compared to ciprofloxacin with MICs of 0.91 µg/mL, 0.12 µg/mL, 0.52 µg/mL, 0.21 µg/mL, 0.63 µg/mL, and 0.15 µg/mL, respectively, while pyrimidine derivatives **5b** and **5i** showed potent antibacterial activities against *P. aeruginosa* compared to ciprofloxacin with MICs of 0.19 µg/mL, 0.13 µg/mL, and 0.22 µg/mL**,** respectively.

On the other hand, the antifungal results shown in Table 1**,** the antifungal activities of ciprofloxacin hybrids **2a-j**, only compound **2c** showed considerable antifungal activity against *C. albicans* compared to ketoconazole with MICs of 3.89 and 2.6 µg/mL, respectively. Moreover, pyrimidine derivatives **5e** displayed potent antifungal activity against *C. albicans* compared to ketoconazole with MICs of 2.51 and 2.60 µg/mL, respectively. Also, hybrids **5a** and **5c** exhibited considerable antifungal activities against *C. albicans* compared to ketoconazole with MICs of 6.23, 2.87, and 2.6 µg/mL, respectively.

Based on the antibacterial and antifungal results recorded in Table 1, it can be deduced that ciprofloxacin hybrids **2a-j** and **5a-i** showed higher antibacterial activity against Gram-positive bacteria (*S. aureus)* than Gram-negative bacteria (*E. coli and P. aeruginosa*). Compound **5b** displayed broad-spectrum antimicrobial activity against all tested strains except *C. albicans*. Concerning chalcone derivatives **2a-j**, it was found that the presence of electron-withdrawing group on the terminal phenyl ring of the chalcone moiety increases the antibacterial activity and *meta* position was preferred. In term of potency, chlorine atom is superior to other halogens for monosubstituted derivatives. Shifting the chorine atom from the *meta* position to the *para* position slightly decreased the antibacterial activity. Also, Replacement of chlorine atom at *para* position with fluorine enhanced the antibacterial activity. Furthermore, introduction of an electron-donating group on the terminal phenyl ring of the chalcone moiety markedly decreased the antibacterial activity. Additionally, regarding SAR studies of ciprofloxacin-pyrimidine hybrids **5a-i** as antibacterial agents, it was found that there is no specific substituent on the phenyl ring directly attached to the pyrimidine core of compounds **5a-j** could determine the activity either electron-donating or electron-withdrawing groups and the enhanced antibacterial activity of some of the pyrimidine derivatives may be attributed to the improvement of the physicochemical properties and consequently enhancing permeability to the bacterial cells. Regarding, SAR studies for antifungal activities of the newly synthesized ciprofloxacin derivatives **1**, **2a-j,** and **5a-i** revealed that the presence of electron-withdrawing group on the terminal phenyl ring of the chalcone moiety was found to improve the antifungal activity and *para* position is optimal for antifungal activity for monosubstituted derivatives. In term of potency, fluorine atom at *para* position was superior to other halogens. Furthermore, introduction of an electron-donating group on the terminal phenyl ring of the chalcone moiety or on the phenyl ring directly attached to the pyrimidine core was found to decrease or abolish the antifungal activity.

#### The Effect of ciprofloxacin hybrids on 2a-c, 2e-f, 5a-c, 5e-i and ciprofloxacin on *S. aureus* DNA gyrase catalytic activity

As antibacterial agents, fluoroquinolones act by targeting and inhibiting DNA gyrase (topo II) which is an essential enzyme involved in the DNA replication process [46–48]. During the processes of DNA replication, gyrase enzyme allows the relaxation of supercoiled DNA by breaking both strands of DNA chain, crossing them over, and then resealing them; hence, the DNA can unwind and replicate [49]. Based on the current design, the inhibitory activity of the most promising ciprofloxacin hybrids **2a-c**, **2e-f**, **5a-c,** and **5e-i** on DNA gyrase as potential molecular target was evaluated utilizing *S. aureus* DNA gyrase Elisa Kit and ciprofloxacin was used as reference. The results of DNA gyrase assay are outlined in Table 2. According to the results in Table 2, compounds **2b** showed potent inhibitory activity against DNA gyrase than the parent ciprofloxacin with IC_50_ of 0.231 µM and 0.323 µM, respectively. Other tested compounds showed good inhibitory activity against DNA gyrase compared to the reference ciprofloxacin with IC_50_ of 1.012–7.592 µM and 0.323 µM, respectively. Hence, it is obvious that the enhanced antibacterial activity of the target ciprofloxacin derivatives could be attributed to inhibition of bacterial DNA gyrase.

**Table 2:** The inhibitory activities of ciprofloxacin hybrids 2a-c, 2e-f, 5a-c, 5e-i and ciprofloxacin on *S. aureus* DNA gyrase

Compound	*S. aureus* DNA gyrase
	IC_50_ ± SD (µM)
**2a**	3.469 ± 0.18
**2b**	0.231 ± 0.01
**2c**	2.995 ± 0.16
**2e**	3.810 ± 0.20
**2f**	1.770 ± 0.09
**5a**	4.546 ± 0.24
**5b**	1.272 ± 0.07
**5c**	1.567 ± 0.08
**5e**	1.012 ± 0.05
**5f**	3.505 ± 0.19
**5g**	7.592 ± 0.40
**5h**	1.456 ± 0.08
**5i**	1.414 ± 0.07
**Ciprofloxacin**	0.323 ± 0.02

### Docking studies on bacterial gyrase enzyme

**Fig. 2:**
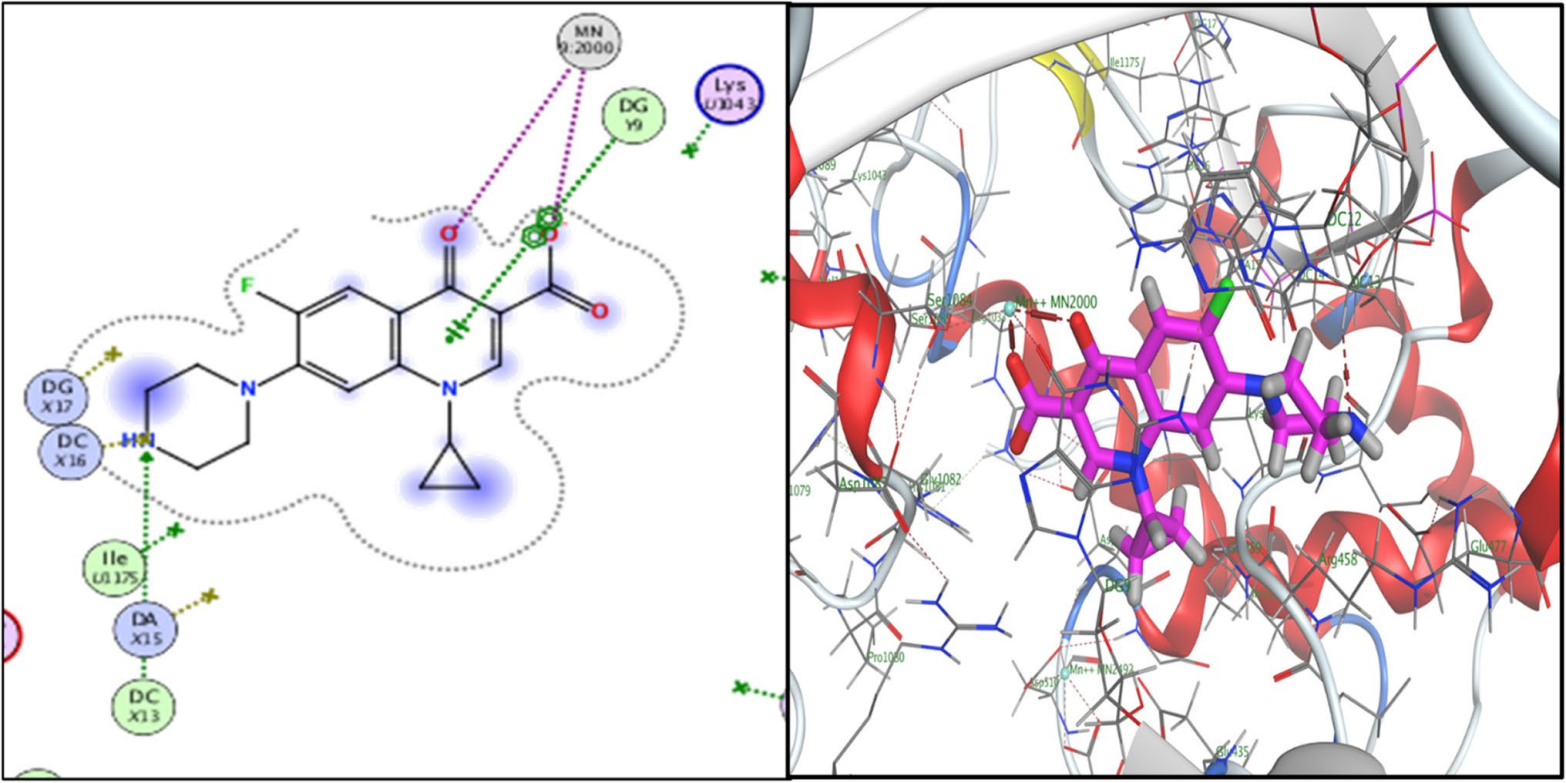
2D- and 3D-binding interactions of ciprofloxacin within gyrase-active site (PDB:**2XCT**)

**Fig. 3:**
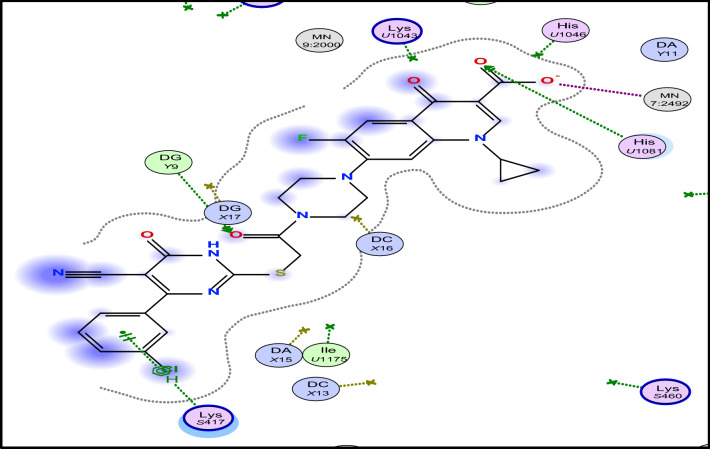
2D-binding interactions of compound **2b** within gyrase-active site (PDB:**2XCT**)

Molecular docking investigation for the most active compounds **2b, 2c, 5a, 5c, 5e, 5h,** and **5i** against *S. aureus* was carried out using Molecular Operating Environment (MOE®) version 2014 to investigate their possible interactions within the active site of gyrase enzyme (PDB: 2XCT). The 3D crystal structure of gyrase enzyme was downloaded from PDB (ID: 2XCT) [50, 51]. Molecular modeling of the co-crystallized ligand (ciprofloxacin) revealed different types of interactions with the active site of gyrase enzyme including a chelation with Mn^+2^ 9:2000, hydrogen bonding with DNA nucleotide base DC X13, hydrophobic interactions, and pi-cationic interaction with DNA nucleotide base DG *Y*9 (Figs. 2, 3). Also, docking studies of ciprofloxacin hybrids **2b, 2c, 5b, 5c, 5e, 5h,** and **5i** revealed that they have the ability to interact with crystal structure of gyrase enzyme via hydrophobic interactions and extra hydrogen bonding interaction with amino acids residues and DNA nucleotide bases (Table 3, Figs. 4, 5, 6, 7 in Supplementary materials) which consistent with their enhanced antibacterial activity against *S. aureus*. All docked compounds had high binding affinity to gyrase enzyme as their energy scores (dG) values are (− 12.8884 to − 10.5550 kcal/mole) higher than that of the co-crystallized ligand ciprofloxacin (dG = − 8.7725 kcal/mole), Table 3**.** The binding-free energies and binding interactions from the major docked poses of ciprofloxacin hybrids **2b, 2c, 5a, 5b, 5c, 5h,** and **5i** are listed in Table 3.

**Table 3:** Types of interactions and energy scores for interaction of ciprofloxacin derivatives **2b**, **2c**, **5a**, **5b, 5c, 5e, 5h, 5i**, and the reference ciprofloxacin within the active site of Bacterial gyrase (PDB: 2XCT)

#	Types of interactions	Energy score Kcal/mole
Ciprofloxacin	• Chelation with Mn^+2^ 9:2000 via the *C*-3 carboxylic group and *C*-4 carbonyl functionality of quinolone moiety • Hydrogen bonding with DNA nucleotide base DC X13 • pi-cationic interaction with DNA nucleotide base DG Y9 • Hydrophobic interactions	− 8.1332
2b	• Hydrogen bond with Mn^+2^ 9:2492 via carbonyl of quinolone moiety • Hydrogen bond with His. 2084 via carbonyl of urea linker • Hydrogen bonding with DNA nucleotide base DG Y9 • pi-cationic interaction with Lys. S417 • Hydrophobic interactions	− 10.0950
2c	• Hydrogen bond with Mn^+2^ 9:2000 via carbonyl of quinolone moiety • Hydrogen bonding with DNA nucleotide base DG Y9 • Hydrophobic interactions	− 9.5058
5a	• Hydrogen bond with Mn^+2^ 7:2492 via carboxylic group of quinolone moiety • Hydrogen bond with His 1034 via carbonyl of carboxylic group • pi-cationic interaction with Arg. 158 Hydrophobic interactions	− 10.0895
5b	• Hydrogen bond with Mn^+2^ 7:2492 via carboxylic group of quinolone moiety • Hydrogen bond with His 1034 via carbonyl of carboxylic group • Hydrogen bonding with DNA nucleotide base DG Y9 • pi-cationic interaction with Lys. S117 • Hydrophobic interactions	− 10.2850
5c	• Hydrogen bond with Mn^+2^ 7:2492 via carboxylic group of quinolone moiety • Hydrogen bond with Glu. S477 and Arg. S158 • pi-cationic interaction with DNA nucleotide base DG Y9 Hydrophobic interactions	− 9.9704
5e	• Chelation with Mn^+2^ 9:2000 via the *C*-3 carboxylic group and *C*-4 carbonyl functionality of quinolone moiety • Hydrogen bonding with DNA nucleotide base DC X12 • Two pi-cationic interaction with DNA nucleotide base DG Y9 Hydrophobic interactions	− 10.3587
**5h**	• Chelation with Mn^+2^ 9:2000 via the *C*-3 carboxylic group and *C*-4 carbonyl functionality of quinolone moiety • Hydrogen bonding with Asn.S476 and Asn. S475 • Two pi-cationic interaction with DNA nucleotide base DG Y9 Hydrophobic interactions	− 10.7105
5i	• Chelation with Mn^+2^ 9:2000 via the *C*-3 carboxylic group and *C*-4 carbonyl functionality of quinolone moiety • Two pi-cationic interaction with DNA nucleotide base DG Y9 Hydrophobic interactions	− 10.9547

### Determination of solubility and lipophilicity

Physicochemical properties of drug molecules are very important parameters that affect biological activity. Therefore, it is necessary to investigate the solubility and lipophilicity of some of the active series of the target compounds **2a-i**. The results of measuring solubility and lipophilicity are outlined in Table 4.

**Table 4:** Solubility pHs of 6.8 and 7.8 at 37 °C (mg/mL) and lipophylicity expressed in (Log *P*_exp_) of synthesized target compounds 2a-i and Cioprofloxacin

Compound #	Solubility mg/mL		Lipophilicity (log p)
	pH 6.8	pH 7.8	
**Cioprofloxacin**	0.12	0.16	− 0.56
**2a**	0.21	0.15	0.24
**2b**	0.16	0.18	0.31
**2c**	0.13	0.15	0.07
**2d**	0.14	0.16	0.42
**2e**	0.16	0.18	0.07
**2f**	0.16	0.17	0.4
**5g**	0.74	0.9	− 0.09
**2j**	0.18	0.15	0.34
**2h**	0.68	0.78	− 0.21
**2i**	0.15	0.19	− 0.06

As shown in Table 4, the solubility studies of compounds **2a-i** in buffer solutions at pH 6.8 and 7.8 and temperature 37 ± 0.5 showed that the solubility values of the tested compounds are variable. The strength of the bond in ion solvates is expected to be more than that between the unionized forms of the molecules and solvent. Hence, solubility of the charged forms of the fluoroquinolone molecules is expected to exceed the solubility of a neutral analog [52]. The fluoroquinolones under study have a basic (N-) and acidic groups, (-COOH) and -CONH). These polar groups in these molecules can participate in formation of hydrogen bonds with the solvent and strong intermolecular interactions.

Partition coefficient of ciprofloxacin hybrids **2a-j** between octanol and buffer solution was studied at temperature of 37 ± 0.5 and pH 7.8 using the isothermal saturation method. Results in Table 4 express the distribution coefficients of drugs in logarithmic scale obtained from the experimental concentrations in organic and water phases (log p). [53, 54]. According to the distribution of fluoroquinolones in octanol/buffer pH 7.4 system, the lipophilicity of the tested compounds decreases in the following order: **2d > 2f > 2j > 2b** > **2a.** Results from a similar study using enrofloxacin molecule [55] showed that a comparatively high lipophilicity could be attributed to its less polar surface area. It is obvious that substitution at piperazinyl N-4 increased lipophilicity of the tested compounds that may be due to decrease in the possibility of zwitter ion formation. It is clear from Table 4 that the distribution coefficients decrease in buffer solutions at lower pH, the cationic species dominate in comparison with pH 7.4 when the zwitterionic forms predominate.

The observation that the fluoroquinolones which are secondary amines have increased partitioning at pH 5 as compared with the tertiary amines further suggests that ion pairing is a possible explanation because it has been observed that the order of ion-pair formation constants of amines is 3° < 2° < 1° [56]. The order of ion-pairing has been attributed to the number of hydrogen atoms available to hydrogen bond with the anion and stabilized the ion pair.

## Conclusion

New ciprofloxacin derivatives **2a-j** and **5a-i** were synthesized and investigated for their antibacterial activity. In general, ciprofloxacin hybrids **2a-j** and **5a-i** showed potent antibacterial activity against Gram-positive bacteria (*S. aureus*) than Gram-negative bacteria (*E. coli* and *P. aeruginosa*). On the other hand, compounds **2c**, **2e**, **5c,** and **5e** revealed comparable antifungal activity to ketoconazole against *candida albicans*. Further investigations showed that some ciprofloxacin hybrids have an inhibitory effect on the catalytic activity of DNA gyrase as potential molecular target. Structure activity relationship of hybrids **2a-j** indicated that the presence of electron-withdrawing group on the terminal phenyl ring of the chalcone moiety increases the antibacterial activity and meta position was preferred, while introduction of an electron-donating group markedly decreases the antibacterial activity. Regarding ciprofloxacin-pyrimidine hybrids **5a-i** as antibacterial agents, it was found that there is no specific substituent on the phenyl ring directly attached to the pyrimidine core of compounds **5a-j** could determine the activity either electron-donating or electron-withdrawing groups. Docking studies against DNA gyrase-active site (PDB: 2XCT) confirmed the ability of compounds **2b**, **2c**, **5a**, **5b**, **5c,** and **5e** to interact with DNA gyrase enzyme.

## Experimental

### Chemistry

#### Materials and methods

All chemical and reagents used for synthetic procedures and biological evaluations were purchased from commercial suppliers. Chemical Reactions were monitored by analytical thin layer chromatography (TLC), using precoated silica gel 60 F245 aluminum plate. Melting points were recorded on a Stuart SMP30 melting point apparatus and were uncorrected. NMR spectra (400 MHz for ^1^H, 100 MHz for ^13^C) were observed in DMSO- *d*_6_ on Bruker AM400 spectrometer with tetramethylsilane as the internal standard. Elemental analyses were recorded on Shimadzu GC/MS-QP5050A, Regional center for Mycology and Biotechnology, Al-Azhar University, Cairo, Egypt. Mass spectra were recorded on Advion compact mass spectrometer (CMS) and reported as mass/charge (m/z), Nawah scientific center for research, Almokattam, Cairo, Egypt.

#### General procedure for synthesis of 7-(4-((4-cinnamoylphenyl)carbamoyl)piperazin-1-yl)-1-cyclopropyl-6-fluoro-4-oxo-1,4-dihydroquinoline-3-carboxylic acid derivatives 2a-j

An equimolar amount of ciprofloxacin derivative 1 (0.492 g, 1 mmol) and the appropriate aromatic aldehyde (1.1 mmol) were dissolved in a minimum amount of ethanol, and aqueous NaOH (2.5 mmol) was added as 60% solution in a dropwise manner. The reaction mixture was stirred in an ice bath for 30 min then at rt for 8–12** h** then the reaction mixture was acidified with acetic acid. The formed precipitate was filtered off and washed thoroughly with cold-distilled water and cold methanol. The product was recrystallized from absolute ethanol [56].

#### General procedure for synthesis of 2-mercapto-6-oxo-4-phenyl-1,6-dihydropyrimidine-5-carbonitrile derivatives 3a-i

To a mixture of ethyl cyanoacetate (1.2 mL, 11.21 mmol), thiourea (0.85 g, 11.21 mmol) and the appropriate aldehyde (10.2 mmol) in 50 mL absolute ethanol, potassium carbonate (1.56 g, 11.21 mmol) was added. The reaction mixture was heated under reflux for 6 h, cooled, poured into 50 mL distilled water, and acidified with few drops of glacial acetic acid to give a precipitate, which was filtered off, dried, and recrystallized from DMF/water to give compounds **8a**-**i** [40, 41]

#### General procedure for synthesis of 7-(4-(2-((5-cyano-6-oxo-4-phenyl-1,6-dihydropyrimidin-2-yl)thio)acetyl)piperazin-1-yl)-1-cyclopropyl-6-fluoro-4-oxo-1,4-dihydroquinoline-3-carboxylic acid derivatives 5a-i

An equimolar mixture of compound **4** (0.30 g, 0.660 mmol) and intermediates **3a-i** (0.73 mmol) in acetonitrile (50 mL), and TEA (0.79 mmol, 0.20 mL) were added. The reaction mixture was heated at reflux for 6–8** h**. The reaction mixture was evaporated to dryness. The residue was crystallized from acetonitrile to afford the target compounds **5a-i** [17].

##### 7-(4-(2-((5-Cyano-6-oxo-4-phenyl-1,6-dihydropyrimidin-2-yl)thio)acetyl)piperazin-1-yl)-1-cyclopropyl-6-fluoro-4-oxo-1,4-dihydroquinoline-3-carboxylic acid 5a

White powder; (0.29 g, 73.12% yield); mp: 237–240 ºC; ^1^H NMR (400 MHz, DMSO-*d*_6_) δ: 1.18–1.22 (2H, m, cyclopropyl-*H*), 1.32–1.33 (2H, m, cyclopropyl-*H*), 3.30–3.38 (4H, m, piperazinyl-*H*), 3.71–3.78 (5H, m, piperazinyl-*H* and cyclopropyl-*H*), 4.42 (2H, s, S-C*H*_2_), 7.53–7.58 (4H, m, Ar–*H* and C8-*H*), 7.93–7.96 (3H, m, Ar–*H* and C5-*H*), 8.68 (1H, s, C2-*H*), 14.29–14.35 (1H, brs, CO–N*H*), 15.15 (1H, brs, COO*H*); ^13^C NMR (100 MHz, DMSO-*d*_6_) δ: 8.07, 34.59, 38.81, 45.46, 49.49, 49.93, 93.43, 106.93, 107.34, 111.37, 111.58 (C-5, d, ^2^*J*_FC*ortho*_ = 23 Hz, C-F), 116.27, 119.32, 121.22, 123.96, 128.99, 132.17, 135.76, 136.87, 139.65, 145.25, 148.56, 153.37 (C-6, d, ^1^*J*_FC*ipso*_ = 250 Hz, C-F), 165.57, 166.33, 174.92; ESI–MS (m/z): Calcd. 600.16, found 599.70 [M-H] ^−^ Anal. Calcd. For C_30_H_25_FN_6_O_5_S: C, 59.99; H, 4.20; N, 13.99; S, 5.34. Found: C, 60.21; H, 4.37; N, 13.85; S, 5.41.

##### 7-(4-(2-((4-(3-Chlorophenyl)-5-cyano-6-oxo-1,6-dihydropyrimidin-2-yl)thio)acetyl)piperazin-1-yl)-1-cyclopropyl-6-fluoro-4-oxo-1,4-dihydroquinoline-3-carboxylic acid 5b

Yellow powder; (0.32 g, 76.23% yield); mp: 248–251 ºC; ^1^H NMR (400 MHz, DMSO-*d*_6_) δ: 1.20–1.26 (2H, m, cyclopropyl-*H*), 1.32–1.33 (2H, m, cyclopropyl-*H*), 3.30–3.38 (4H, m, piperazinyl-*H*), 3.72–3.81 (5H, m, piperazinyl-*H* and cyclopropyl-*H*), 4.37(2H, s, S-C*H*_2_), 7.53 (1H, d, *J*_HF*meta*_ = 8 Hz, C8-*H*), 7.55–7.59 (2H, m, Ar–*H*), 7.87–7.89 (2H, m, Ar–*H*), 7.92 (1H, d, *J*_HF*ortho*_ = 12 Hz, C5-*H*), 8.68 (1H, s, C2-*H*), 11.98 (1H, brs, CO–N*H*), 15.15 (1H, brs, COO*H*); ^13^C NMR (100 MHz, DMSO-*d*_6_) δ: 8.07, 34.23, 36.34, 45.55, 49.46, 49.97, 93.35 106.92, 107.34, 111.54 (C-5, d, ^2^*J*_FC*ortho*_ = 23 Hz, C-F), 112.66, 116.60, 127.71, 128.71, 130.86, 131.59, 131.76, 133.75, 138.09, 138.97, 139.64, 145.15, 148.54, 153.41 (C-6, d, ^1^*J*_FC*ipso*_ = 24**5h**z, C-F), 165.84, 166.00, 166.34, 176.86; ESI–MS (m/z): Calcd. 634.12, found 633.07 [M-H] ^−^. Anal. Calcd. For C_30_H_24_ClFN_6_O_5_S: C, 56.74; H, 3.81; N, 13.23; S, 5.05. Found: C, 56.86; H, 3.94; N, 13.49; S, 5.12.

##### 7-(4-(2-((4-(4-Chlorophenyl)-5-cyano-6-oxo-1,6-dihydropyrimidin-2-yl)thio)acetyl)piperazin-1-yl)-1-cyclopropyl-6-fluoro-4-oxo-1,4-dihydroquinoline-3-carboxylic acid 5c

Yellowish white powder; (0.31 g, 73.98% yield); mp: 253–256 ºC; ^1^H NMR (400 MHz, DMSO-*d*_6_) δ: 1.20–1.22 (2H, m, cyclopropyl-*H*), 1.32–1.36 (2H, m, cyclopropyl-*H*), 3.34–3.39 (4H, m, piperazinyl-*H*), 3.72–3.85 (5H, m, piperazinyl-*H* and cyclopropyl-*H*), 4.22 (2H, s, S-C*H*_2_), 7.55 (2H, d, *J* = 8 Hz, Ar–*H*), 7.58 (1H, d, *J*_HF*meta*_ = 8 Hz, C8-*H*), 7.87 (2H, d, *J* = 8 Hz, Ar–*H*), 7.93 (1H, d, *J*_HF*ortho*_ = 13 Hz, C5-*H*), 8.68 (1H, s, C2-*H*), 15.10 (1H, brs, COO*H*); ^13^C NMR (100 MHz, DMSO-*d*_6_) δ: 8.02, 33.32, 36.32, 45.62, 49.14, 52.70, 91.27, 107.20, 111.75 (C-5, d, ^2^*J*_FC*ortho*_ = 22 Hz, C-F), 114.93, 120.26, 125.25, 126.28, 130.87, 131.71, 133.10, 137.75, 139.93, 142.07, 145.69, 150.52, 153.67 (C-6, d, ^1^*J*_FC*ipso*_ = 2456 hz, C-F), 162.02, 164.55, 166.46, 176.38; ESI–MS (m/z): Calcd. 634.12, found 633.90 [M-H] ^−^. Anal. Calcd. For C_30_H_24_ClFN_6_O_5_S: C, 56.74; H, 3.81; N, 13.23; S, 5.05. Found: C, 57.02; H, 3.94; N, 13.51; S, 5.21.

##### 7-(4-(2-((4-(4-Bromophenyl)-5-cyano-6-oxo-1,6-dihydropyrimidin-2-yl)thio)acetyl)piperazin-1-yl)-1-cyclopropyl-6-fluoro-4-oxo-1,4-dihydroquinoline-3-carboxylic acid 5d

Yellow powder; (0.3**5g**, 77.89% yield); mp: 284–285 ºC; ^1^H NMR (400 MHz, DMSO-*d*_6_) δ: 1.20–1.27 (2H, m, cyclopropyl-*H*), 1.33–1.35 (2H, m, cyclopropyl-*H*), 3.36–3.41 (4H, m, piperazinyl-*H*), 3.71–3.83 (5H, m, piperazinyl-*H* and cyclopropyl-*H*), 4.40 (2H, s, S-C*H*_2_), 7.53 (1H, d, *J*_HF*meta*_ = 8 Hz, C8-*H*), 7.73 (2H, d, *J* = 8 Hz, Ar–*H*), 7.86 (2H, d, *J* = 8 Hz, Ar–*H*), 7.93 (1H, d, *J*_HF*ortho*_ = 16 hz, C5-*H*), 8.68 (1H, s, C2-*H*), 15.14 (1H, brs, COO*H*); ^13^C NMR (100 MHz, DMSO-*d*_6_) δ: 8.06, 34.40, 36.33, 38.21, 44.04, 45.48, 49.36, 91.64, 107.35, 111.58 (C-5, d, ^2^*J*_FC*ortho*_ = 23 Hz, C-F), 114.41, 119.32, 121.23, 125.35, 131.18, 137.56, 139.21, 139.64, 145.28, 148.55, 151.15, 153,51 (C-6, d, ^1^*J*_FC*ipso*_ = 24**5h**z, C-F), 158.64, 162.54, 165.44, 166.33, 177.06; ESI–MS (m/z): Calcd. 678.07, found 677.60 [M-H] ^−^. Anal. Calcd. For C_30_H_24_BrFN_6_O_5_S: C, 53.03; H, 3.56; N, 12.37; S, 4.72. Found: C, 53.29; H, 3.52; N, 12.09; S, 4.81.

##### 7-(4-(2-((4-(4-Fluorophenyl)-5-cyano-6-oxo-1,6-dihydropyrimidin-2-yl)thio)acetyl)piperazin-1-yl)-1-cyclopropyl-6-fluoro-4-oxo-1,4-dihydroquinoline-3-carboxylic acid 5e

Pale yellow powder; (0.30 g, 73.11% yield); mp: 242–245 ºC; ^1^H NMR (400 MHz, DMSO-*d*_6_) δ: 1.19–1.21 (2H, m, cyclopropyl-*H*), 1.32–1.33 (2H, m, cyclopropyl-*H*), 3.35–3.42 (4H, m, piperazinyl-*H*), 3.70–3.84 (5H, m, piperazinyl-*H* and cyclopropyl-*H*), 4.44 (2H, s, S-C*H*_2_), 7.38 (2H, t, *J*_HF*ortho*_ = 8 Hz, Ar–*H*), 7.52 (1H, d, *J* = 8 Hz, C8-*H*), 7.92 (1H, d, *J*_HF*ortho*_ = 12 Hz, C5-*H*) 8.02 (2H, t, *J*_F_ = 8 Hz, Ar–*H*), 8.67 (1H, s, C2-*H*), 15.14 (1H, brs, COO*H*); ^13^C NMR (100 MHz, DMSO-*d*_6_) δ: 8.05, 34.74, 36.34, 44.72, 45.51, 49.62, 97.07, 107.01, 107.33, 111.53 (C-5, d, ^2^*J*_FC*ortho*_ = 23 Hz, C-F), 115.68 (C_Ar_, d, ^2^*J*_FC*ortho*_ = 21 Hz, C-F), 119.39, 131.30, 133.50, 138.96, 139.65, 145.81, 146.64, 148.46, 148.63, 153.42 (C-6, d, ^1^*J*_FC*ipso*_ = 24**5h**z, C-F), 155.21, 162.56 (C_Ar_, d, ^*1*^*J*_CF*ipso*_ = 286 hz, C-F), 165.00, 166.34, 176.86; ESI–MS (m/z): Calcd. 618.15, found 617.70 [M-H] ^−^. Anal. Calcd. For C_30_H_24_F_2_N_6_O_5_S: C, 58.25; H, 3.91; N, 13.59; S, 5.18. Found: C, 58.43; H, 4.12; N, 13.75; S, 5.34.

##### 7-(4-(2-((5-Cyano-6-oxo-4-(*p*-tolyl)-1,6-dihydropyrimidin-2-yl)thio)acetylpiperazin-1-yl)-1-cyclopropyl-6-fluoro-4-oxo-1,4-dihydroquinoline-3-carboxylic acid 10f -methyl 5f.

Pale yellow powder; (0.33 g, 80.95% yield); mp: 264–267 ºC; ^1^H NMR (400 MHz, DMSO-*d*_6_) δ: H-NMR (400 MHz, DMSO-*d*_6_) δ 1.20–1.26 (2H, m, cyclopropyl-*H*), 1.33–1.34 (2H, m, cyclopropyl-*H*), 2.30 ( 3H, s, CH_3_) 3.31–3.37 (4H, m, piperazinyl-*H*), 3.72–3.81 (5H, m, piperazinyl-*H* and cyclopropyl-*H*), 4.36 (2H, s, S-C*H*_2_), 7.31 (2H, d, *J* = 8 Hz, Ar–*H*), 7.55 (1H, d, *J*_HF*meta*_ = 8 Hz, C8-*H*), 7.82 (2H, d, *J* = 8 Hz, Ar–*H*), 7.92 (1H, d, *J*_HF*ortho*_ = 12 Hz, C5-*H*), 8.67 (1H, s, C2-*H*), 13.62 (1H, brs, CO–N*H*), 15.13 (1H, brs, COO*H*); ^13^C NMR (100 MHz, DMSO-*d*_6_) δ: 8.06, 20.80, 34.50, 36.33, 44.05, 49.67, 50.45, 92.77, 107.34, 108.72, 111.55 (C-5, d, ^2^*J*_FC*ortho*_ = 23 Hz, C-F), 112.29, 119.10, 123.40, 128.30, 131.18, 134.44, 138.04, 138.99, 140.20, 146.31, 148.54, 153.40 (C-6, d, ^1^*J*_FC*ipso*_ = 242 Hz, C-F), 161.74, 165.73, 166.31, 176.81; ESI–MS (m/z): Calcd. 614.18, found 613.80 [M-H] ^−^. Anal. Calcd. For C_31_H_27_FN_6_O_5_S: C, 60.58; H, 4.43; N, 13.67; S, 5.22. Found: C, 60.31; H, 4.70; N, 13.90; S, 5.37.

##### 7-(4-(2-((5-Cyano-4-(4-methoxyphenyl)-6-oxo-1,6-dihydropyrimidin-2-yl)thio)acetyl)piperazin-1-yl)-1-cyclopropyl-6-fluoro-4-oxo-1,4-dihydroquinoline-3-carboxylic acid 5 g

Pale yellow powder; (0.28 g, 67.15% yield); mp: 239–242 ºC; ^1^H NMR (400 MHz, DMSO-*d*_6_) δ: 1.20–1.22 (2H, m, cyclopropyl-*H*), 1.31–1.33 (2H, m, cyclopropyl-*H*), 3.35–3.40 (4H, m, piperazinyl-*H*) 3.71–3.76 (4H, m, piperazinyl-*H*), 3.78–383 (4H, m, cyclopropyl-*H* and O-C*H*_3_), 4.42 (2H, s, S-C*H*_2_), 7.52–7.57 (3H, m, C8*-H* and Ar–*H*), 7.92–7.95 (3H, m, C5-*H* and Ar–*H*), 8.68 (1H, s, C2-*H*), 13.75 (1H, brs, CO–N*H*), 15.15 (1H, brs, COO*H*); ^13^C NMR (100 MHz, DMSO-*d*_6_) δ: 8.01, 35.37, 36.28, 44.50, 49.83, 52.36, 55.36, 92.76 106.87, 107.20, 111.52 (C-5, d, ^2^*J*_FC*ortho*_ = 23 Hz, C-F), 120.19, 122.26, 126.45, 129.43, 130.94, 131.29, 135.06, 137.43, 139.65, 145.02, 148.46, 149.92, 153.39 (C-6, d, ^1^*J*_FC*ipso*_ = 246 hz, C-F), 165.98, 166.55, 175.76; ESI–MS (m/z): Calcd. 630.17, found 629.60 [M-H] ^−^. Anal. Calcd. For C_31_H_27_FN_6_O_6_S: C, 59.04; H, 4.32; N, 13.33; S, 5.08. Found: C, 59.31; H, 4.50; N, 13.54; S, 5.17.

##### 7-(4-(2-((5-Cyano-4-(3,4-dimethoxyphenyl)-6-oxo-1,6-dihydropyrimidin-2-yl)thio)acetyl)piperazin-1-yl)-1-cyclopropyl-6-fluoro-4-oxo-1,4-dihydroquinoline-3-carboxylic acid 5h.

Pale yellow powder; (0.2**5g**, 57.00% yield); mp: 266–269 ºC; ^1^H NMR (400 MHz, DMSO-*d*_6_) δ: 1.17–1.21 (2H, m, cyclopropyl-*H*), 1.30–1.33 (2H, m, cyclopropyl-*H*), 3.29–3.37 (4H, m, piperazinyl-*H*), 3.71–3.76 (7H, m, piperazinyl-*H* and O-C*H*_3_), 3.80–3.85 (4H, m, cyclopropyl-*H* and O-C*H*_3_), 4.47 (2H, s, S-C*H*_2_), 7.08 (1H, d, *J* = 8 Hz, Ar–*H*), 7.53 (1H, d, *J*_HF*meta*_ = 8 Hz, C8-*H*), 7.64–7.67 (2H, m, Ar–*H*),) 7.93 (1H, d, *J*_HF*ortho*_ = 16 hz, C5-*H*), 8.68 (1H, s, C2-*H*), 13.92 (1H, brs, CO–N*H*), 15.14 (1H, brs, COO*H*); ^13^C NMR (100 MHz, DMSO-*d*_6_) δ: 8.05, 34.66, 36.30, 41.93, 45.53, 49.45, 56.21, 57.29, 91.77, 107.34, 111.55 (C-5, d, ^2^*J*_FC*ortho*_ = 23 Hz, C-F), 111.81, 112.93, 117.01, 119.37, 123.10, 127.86, 129.53, 132.41, 139.62, 145.16, 145.25, 148.52, 148.82, 152.12, 153.17 (C-6, d, ^1^*J*_FC*ipso*_ = 246 hz, C-F), 161.36, 165.77, 166.35, 176.85; ESI–MS (m/z): Calcd. 660.18, found 659.60 [M-H] ^−^**.** Anal. Calcd. For C_32_H_29_FN_6_O_7_S: C, 58.17; H, 4.42; N, 12.72; S, 4.85. Found: C, 58.29; H, 4.31; N, 12.95; S, 4.72.

##### 7-(4-(2-((5-Cyano-6-oxo-4-(3,4,5-trimethoxyphenyl)-1,6-dihydropyrimidin-2-yl) thio)acetyl)piperazin-1-yl)-1-cyclopropyl-6-fluoro-4-oxo-1,4-dihydroquinoline-3-carboxylic acid 5i.

Pale yellow powder; (0.23 g, 50. % yield); mp: 284-285ºC; ^1^H NMR (400 MHz, DMSO-*d*_6_) δ: 1.20–1.27 (2H, m, cyclopropyl-*H*), 1.32–1.34 (2H, m, cyclopropyl-*H*), 3.30–3.38 (4H, m, piperazinyl-*H*), 3.72 (3H, s, OC*H*_3_), 3.74–3.83 (5H, m, piperazinyl-*H* and cyclopropyl-*H*), 3.86 (6H, s, OC*H*_3_), 4.52 (2H, s, S-C*H*_2_), 7.36 (2H, s, Ar–*H*), 7.53 (1H, d, *J*_HF*meta*_ = 8 Hz, C8-*H*) 7.92 (1H, d, *J*_HF*ortho*_ = 12 Hz, C5-*H*), 8.68 (1H, s, C2-*H*), 11.77 (1H, brs, CO–N*H*), 15.14 (1H, brs, COO*H*); ^13^C NMR (100 MHz, DMSO-*d*_6_) δ: 8.06, 34.98, 36.29, 41.89, 45.53, 49.44, 49.78, 56.70, 60.67, 92.81, 106.89, 107.47, 111.51 (C-5, d, ^2^*J*_FC*ortho*_ = 23 Hz, C-F), 116.61, 119.30, 126.54, 130.48, 134.78, 137.09, 139.58, 141.28, 145.14, 148.47, 152.09, 153.82 (C-6, d, ^1^*J*_FC*ipso*_ = 257 Hz, C-F), 161.74, 165.73, 166.31, 176.81; ESI–MS (m/z): Calcd. 690.19, found 689.60 [M-H] ^−^. Anal. Calcd. For C_33_H_31_FN_6_O_8_S: C, 57.38; H, 4.52; N, 12.17; S, 4.64. Found: C, 57.56; H, 4.68; N, 12.34; S, 4.80.

### Biology

#### Screening of the antimicrobial activity

##### Screening of the antibacterial activity.

The antibacterial activity of compounds **1**, **2a-j**, and **5a-i** and ciprofloxacin were determined according to the standard agar cup diffusion method [45] at Deraya University, Faculty of Pharmacy, Department of Microbiology and Faculty of Postgraduate Studies for Advanced Sciences, Beni-Suef University, Beni-Suef, Egypt.

*Microbial strains and culture conditions* Three bacterial species representing both Gram-positive and Gram-negative strains and were used to test the antibacterial activity of the newly synthesized ciprofloxacin derivatives. Standard strains of *Staphylococcus aureus* (ATCC 6538), *Pseudomonas aeruginosa* (ATCC 10,145), and *Escherichia coli* (ATCC 8739) were obtained from microbiological resource center, Faculty of Agriculture, Ain Shams University, Cairo, Egypt. All isolates were maintained at -70ºC in Trypticase Soya Broth (TSB, Becton, and Dickinson) with 10% glycerol. Prior to inoculation, all isolates were subcultured at 37ºC for 24 h on Trypticase Soya Agar (TSA, Becton, and Dickinson) and TSB, respectively.

*Determination of the minimum inhibitory concentration (MIC)*From all the tested bacteria 0.5 mL of 1 × 108 CFU/mL (0.5 McFarland turbidity) were plated in sterile petri dishes, then 20 mL of Mueller Hinton Agar media (Oxoid) was added to each petri dish. The plates were rotated slowly to ensure uniform distribution of the microorganisms and then allowed to solidify on a flat surface. After solidification, four equidistant and circular wells of 10 mm diameter were carefully punched using a sterile cork bore. Twofold serial dilutions of the tested compounds using DMSO were performed. An equal volume of 100µL of each dilution was applied separately to each well in three replicates using a micropipette. All plates were incubated at 37 ºC for 24 h. The inhibition zones were measured, and their average was calculated. The MIC was calculated by plotting the natural logarithm of the concentration of each dilution of the tested compounds against the square of zones of inhibition, and a regression line was drawn through the points then the antilogarithm of the intercept on the logarithm of concentration axis gave the MIC value [57].

##### Screening of antifungal activity.

The antifungal activity of compounds **1**, **2a-j,** and **5a-i** and ketoconazole were determined according to the agar cup diffusion method [45, 58] at Deraya University, Faculty of Pharmacy, Department of Microbiology.

*Fungal strains and culture conditions. Candida albicans* was used for screening of the antifungal activity of the newly synthesized ciprofloxacin derivatives. Standard strain of *Candida albicans* (ATCC 10,231) was obtained from microbiological resource center, Faculty of Agriculture, Ain Shams University, Cairo, Egypt. The isolate was maintained at -70ºC in Trypticase Soya Broth (TSB, Becton, and Dickinson) with 10% glycerol. Prior to inoculation, the isolate was subcultured at 37ºC for 24 h on Trypticase Soya Agar (TSA, Becton, and Dickinson) and TSB, respectively.

*Determination of the minimum inhibitory concentration (MIC)*From the tested *Candida albicans,* 0.5 mL of 1 × 108 CFU/mL (0.5 McFarland turbidity) was plated in sterile petri dishes, then 20 mL of Sabouraud agar was added to each petri dish. The plates were rotated slowly to ensure uniform distribution of the microorganisms and then allowed to solidify on a flat surface. After solidification, four equidistant and circular wells of 10 mm diameter were carefully punched using a sterile cork bore. Twofold serial dilutions of the tested compounds using DMSO were performed. An equal volume of 100µL of each dilution was applied separately to each well in three replicates using a micropipette. All plates were incubated at 37 ºC for 24 h. The inhibition zones were measured, and their average was calculated. The MIC was calculated by plotting the natural logarithm of the concentration of each dilution of the tested compounds against the square of zones of inhibition and a regression line was drawn through the points then the antilogarithm of the intercept on the logarithm of concentration axis gave the MIC value [45, 58].

##### *Staphylococcus aureus* DNA Gyrase Supercoiling Assay

*Staphylococcus aureus* DNA gyrase assay was performed according to established protocols obtained from inspirals (Cat. No. SAS4001) The new compounds and ciprofloxacin were dissolved in DMSO and serially diluted at concentrations of 100, 10, 1, and 0.1 μM, and then assayed in reaction mixtures in three different replicate runs.

*Staphylococcus aureus* DNA gyrase was incubated at 37 °C for 30 min in a total reaction volume of 30 μl containing 40 mM HEPES. KOH (pH 7.6), 10 mM magnesium acetate, 10 mM DTT, 2 mM ATP, 500 mM potassium glutamate, 0.05 mg/ml albumin and Relaxed pBR322. DNA gyrase supercoiling reactions catalyzed by *S. aureus* gyrase, Stop reaction by adding 30 μL of STEB and 30 μL of chloroform/isoamyl alcohol (v:v, 24:1),and then Vortex briefly ~ 5 secs and centrifuge for 1 min. after which 20 µL of this was loaded on a 1% agarose gel that was then run at ~ 75 V for approximately 2 h. The gel was stained by (0.5 mg/L) ethidium bromide in water. Fluorescent images were taken at a wavelength of 300 nm on a UV transilluminator imaging system. The fluorescence intensity of the supercoiled plasmid reaction product was quantitated using ImagQuant software (Molecular Dynamics). The results as IC50 values (concentration of the tested compound that leads to50% inhibition of enzyme activity) for all samples were determined by nonlinear regression analysis inGraphPad Prism.

### Docking studies on bacterial gyrase enzymes

All the compounds were drawn in Chem Draw professional (ver. 2015), converted to smiles, transferred to Molecular Operating Environment (MOE 2014) program. Hydrogens were added, and finally the energy of the docked structures was minimized using MMF94FX forcefield with a gradient RMS of 0.001 kcal/mol. Bacterial gyrase in complex with DNA and ciprofloxacin (2XCT) protein was downloaded from the RCSB Protein Data Bank (https://www.rcsb.org/). Bacterial gyrase in complex with DNA and ciprofloxacin X-ray crystal structure with 3.35 Å resolution (PDB ID: 2XCT) was used for docking studies. The protein was prepared by using the MOE quickprep protocol. The ligands were then docked in the binding‏ ‏site using the triangle matcher placement method. The number of generated poses was set for 10 for each ligand, and default settings were employed for other parameters. Refinement was carried out using Forcefield and‏ ‏scored using the affinity ΔG scoring system. To validate the docking study at the 2XCT-active site, the co-crystallized ligand was re-docked into the binding site using the same set of parameters as described above. The resulting docking poses were visually inspected, and the poses of the lowest binding-free energy value and with the best hydrophobic, H-bonding, and electrostatic interactions within the binding pocket of target protein.

### Determination of solubility and lipophilicity

#### UV spectrophotometric scanning of drug

A sample of drug was accurately weighed and dissolved in 100 ml of distilled water to prepare 100 mcg/ml stock solutions. Appropriate dilution of the stock solution with phosphate buffer pH 6.8 and 7.8 was then made to prepare a working solution of 40mcg/ml. The absorbance of the drug in the working solution was scanned in the ultraviolet region (200–400 nm) to determine the wavelength of maximum absorbance (λ_max_ = 272 nm).

#### Construction of UV calibration curve

After determination of λ max of drug, a calibration curve was constructed by preparing solutions containing different concentrations of (1–5 mcg/ml) from stock solution after appropriate dilution with phosphate buffer pH 6.8. The UV absorbance of the prepared sample solutions were measured at the predetermined λ max using phosphate buffer pH 6.8 as a blank.

#### Solubility determinations

Aqueous solubility of the complexes was measured as a function of pH. An excess of each derivative was placed into suitable stoppered containers. Seven of these containers (in triplicate) were added with variable volumes of phosphate buffer to obtain pH values of 6.8 and 7.8. The samples were immersed in a water bath thermostatized at 37 ± 1^◦^C and 100 rpm and periodically shaken for 48 h. Once the equilibrium was reached, the pH of the supernatant was regarded. Aliquots of the filtrate properly diluted with suitable buffer were analyzed by UV spectrophotometry (Shimadzu *UV A-160)* at the maximum wavelengths (λ _max_ 272 nm) [57, 59].

#### Determination of partition coefficients

The n-octanol/water apparent partition coefficient at the isoelectric point (or distribution constant) was measured by using the traditional shake-flask technique. The pH of the aqueous buffer was first adjusted to isoelectric point of the compound. The n-octanol and aqueous phases were then mutually saturated before the measurement. The compound was dissolved in aqueous buffer solution and the solution was equilibrated with n-octanol for 1 h. The aqueous concentration to n-octanol concentration ratio (C_aq_/C_oct_) was 1:1. The samples were agitated for **5h** at water bath shaker thermostatized at 25 ± 1^◦^C and 100 rpm. The concentration of the solute was determined in the aqueous and organic phases at the isoelectric point by UV spectroscopy the log p value was calculated as follows:

$$\mathrm{log}p=\frac{[conc.aq]}{[conc. oct]}.$$

## Supplementary Information

Below is the link to the electronic supplementary material.

Supplementary file1 (DOCX 2138 KB)
