# Supplementary material for: Synthesis and molecular docking of new N4-piperazinyl ciprofloxacin hybrids as antimicrobial DNA gyrase inhibitors
Source: Mol Divers. 2022 Sep 24;27(4):1751–65. doi: 10.1007/s11030-022-10528-z (PMC10415461; doi:10.1007/s11030-022-10528-z)
Supplement: Supplementary file 1 — Supplementary file1 (DOCX 2138 KB) [file 11030_2022_10528_MOESM1_ESM.docx]

**Supplementary data**

**Synthesis and molecular docking of new *N*4-piperazinyl ciprofloxacin hybrids as antimicrobial DNA gyrase inhibitors**

**Hamada H.H. Mohammed^1,2,3*^, Doaa Mohamed Elrooby Ali^4^, Mohamed Badr^5^, Ahmed G.K. Habib^6^, Abobakr Mohamed Mahmoud^7^, Sarah M. Farhan^7^, Shimaa Salah Hassan Abd El Gany^7^, Soad A. Mohamad ^8^ , Alaa M. Hayallah^9,10^,** [**Samar H Abbas**](https://www.researchgate.net/profile/Samar_Abbas6?_sg%5B0%5D=mkPHDtm-YANKygOyzayqFrjB3TeL8vEHhPoI5d0hRegJDi18-96m7mB75kJExYHROIl0Qk0.FFpfqe6owJHXeo6u7rcAFPhhI2_xdllgqU8WgE-d_34UjHOBsc1Wu3WgrY2Gksn08gkHDUzt7bPn4TpO2PPV2Q&_sg%5B1%5D=VhRrT7PATBq1bQisAs0zLLUBQrQxkYSNJvp_QHkFnmtDTVACy61srBT9c5r0Sdp7PSICZR4pb4CQHWPs.UOmMD95eiSa04RnTM70npebwuV9Ml-hMcWezDC2uJLxfT47d2vVC_XmKF2RG4vNAG5Sd1IAg81JzwzxV46SM-g)**^2^,** [**Gamal El-Din A Abuo-Rahma**](https://www.researchgate.net/profile/Gamal_El-Din_Abuo-Rahma?_sg%5B0%5D=mkPHDtm-YANKygOyzayqFrjB3TeL8vEHhPoI5d0hRegJDi18-96m7mB75kJExYHROIl0Qk0.FFpfqe6owJHXeo6u7rcAFPhhI2_xdllgqU8WgE-d_34UjHOBsc1Wu3WgrY2Gksn08gkHDUzt7bPn4TpO2PPV2Q&_sg%5B1%5D=VhRrT7PATBq1bQisAs0zLLUBQrQxkYSNJvp_QHkFnmtDTVACy61srBT9c5r0Sdp7PSICZR4pb4CQHWPs.UOmMD95eiSa04RnTM70npebwuV9Ml-hMcWezDC2uJLxfT47d2vVC_XmKF2RG4vNAG5Sd1IAg81JzwzxV46SM-g)**^2,3*^**

^1^Department of Pharmaceutical Chemistry, Faculty of Pharmacy, Sohag University, Sohag 82524, Egypt

^2^Department of Medicinal Chemistry, Faculty of Pharmacy, Minia University, 61519-Minia, Egypt

^3^Department of Pharmaceutical Chemistry, Faculty of Pharmacy, Deraya University, New, Minia City 61768, Egypt

^4^Department of Biochemistry, Faculty of Pharmacy, Sohag University, Sohag 82524, Egypt

^5^Department of Biochemistry, Faculty of Pharmacy, Menoufia University, Menoufia, Egypt

^6^Department of Biotechnology and Life Sciences, Faculty of Postgraduate Studies for Advanced Sciences, Beni-Suef University, Beni-Suef, Egypt

^7^Department of Microbiology and immunology, Faculty of Pharmacy, Deraya University, New Minia City 61768, Egypt

^8^Department of of Pharmaceutics and clinical pharmacy, Faculty of Pharmacy, Deraya University, New-Minia 61768, Minya, Egypt

^9^Pharmaceutical Organic Chemistry Department, Faculty of Pharmacy, Assiut University, 71526, Egypt

^10^Pharmaceutical Chemistry Department, Faculty of Pharmacy, Sphinx University, New Assiut, Egypt

**To whom correspondence should be addressed:*

*G. E-D. A. Abuo -Rahma: [gamal.aborahama@mu.edu.eg](mailto:gamal.aborahama@mu.edu.eg), +201003069431

*Hamada. H.H. Mohammed: [hamada.hashem@pharm.sohag.edu.eg](mailto:hamada.hashem@pharm.sohag.edu.eg), +201002261392

**H-NMR of compound 5a**


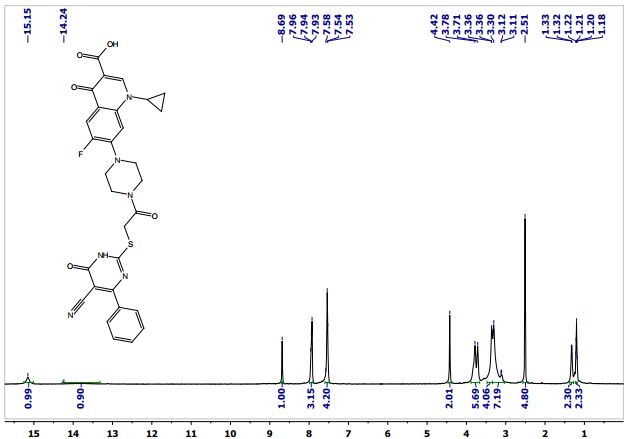


**13C-NMR for compound 5a**


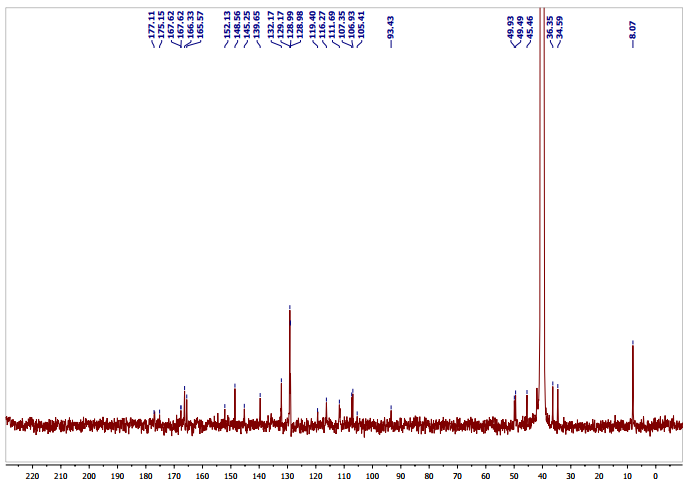


**Mass spectra of compound 5a**


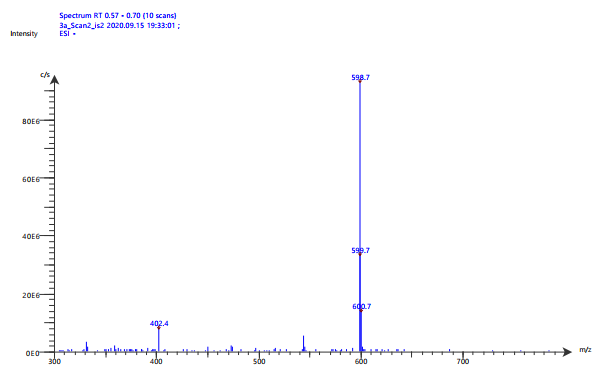


**H-NMR for compound 5b**


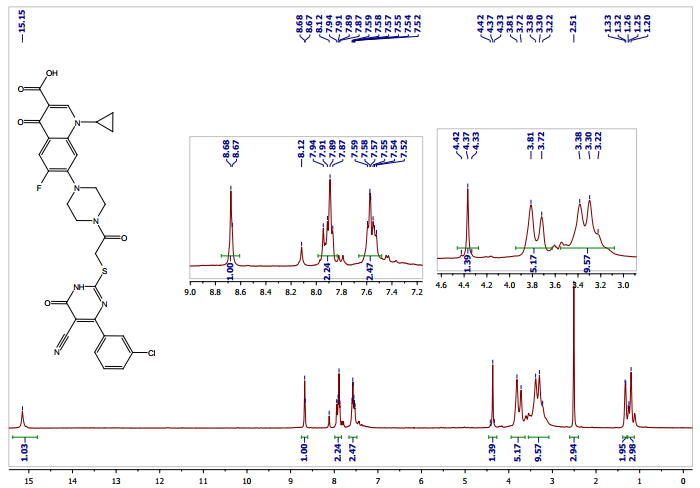


**13C-NMR for compound 5b**


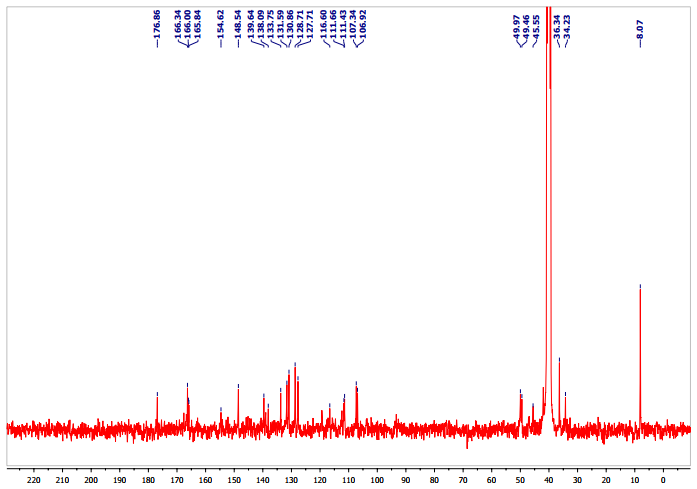


**Mass spectra of compound 5b**


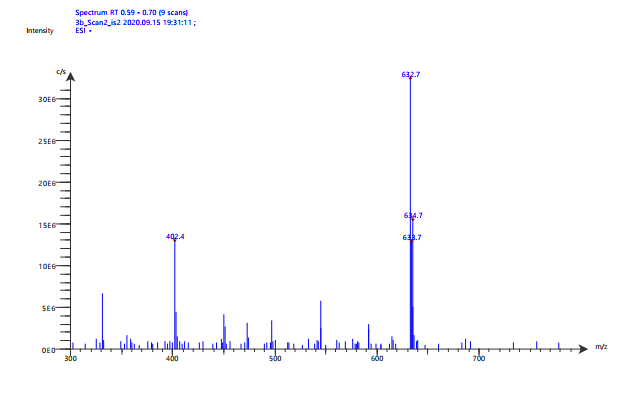


**H-NMR for compound 5C**


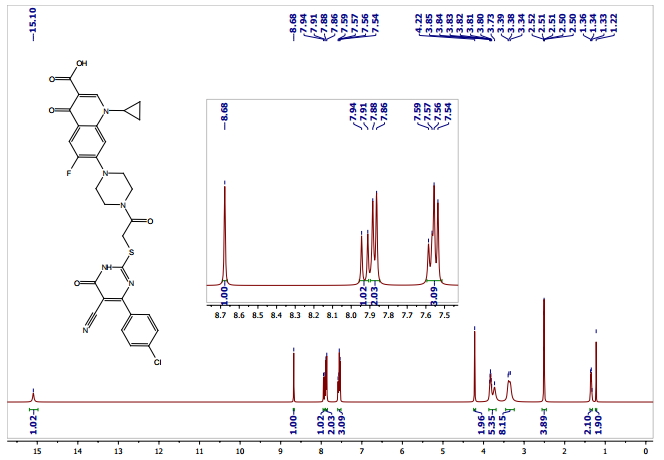


**13C-NMR for compound 5c**


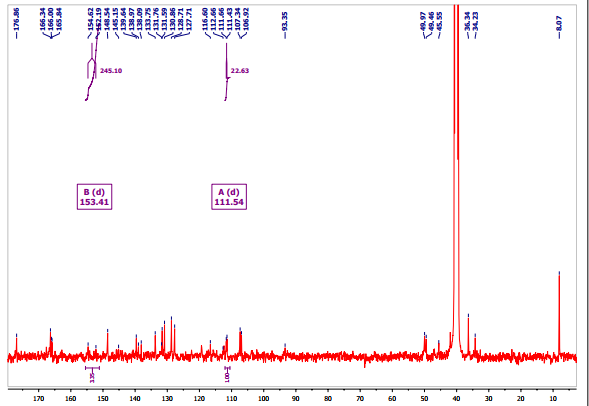


**Mass spectra of compound 5c**


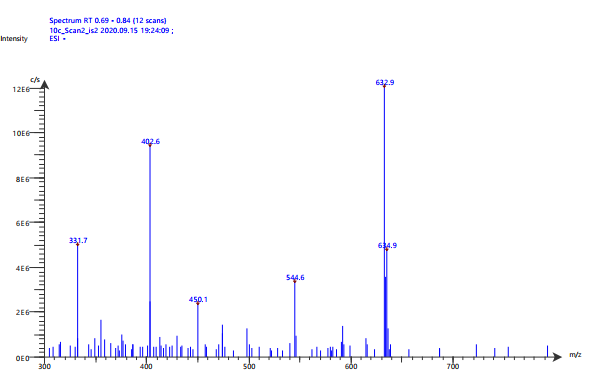


**H-NMR for compound 5d**


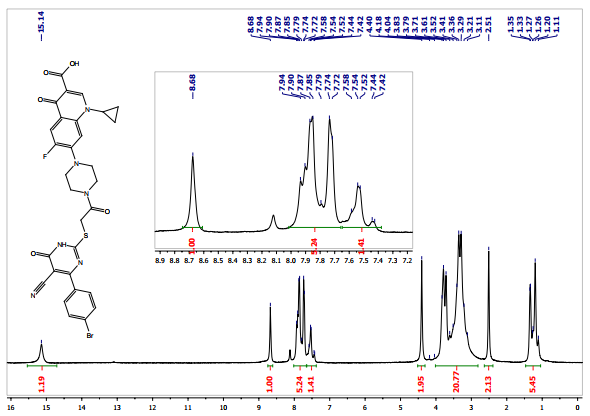


**13c-NMR for compound 5d**


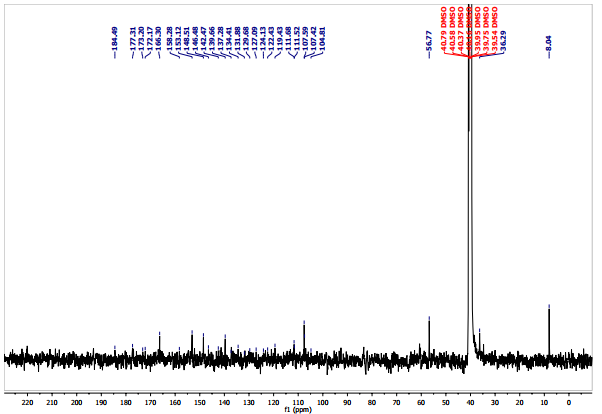


**Mass spectra of compound 5d**


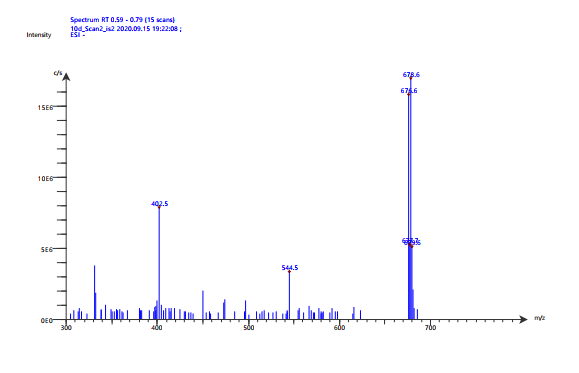


**H-NMR for compound 5e**


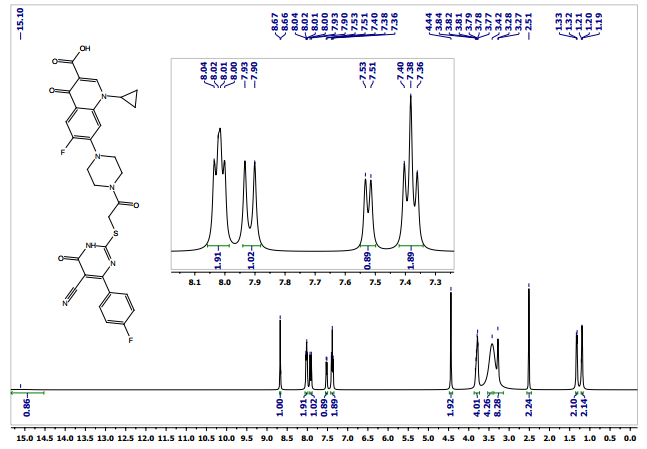


**13c-NMR for compound 5e**


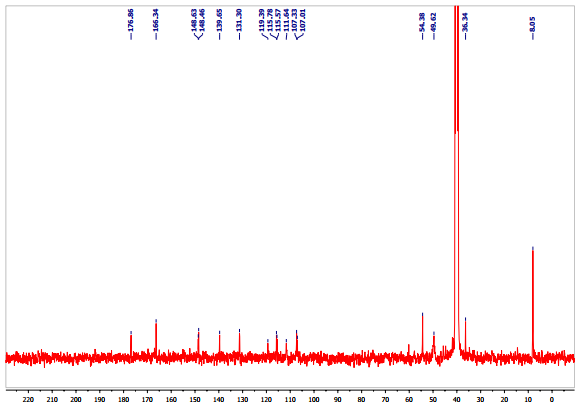


**Mass spectra of compound 5e**


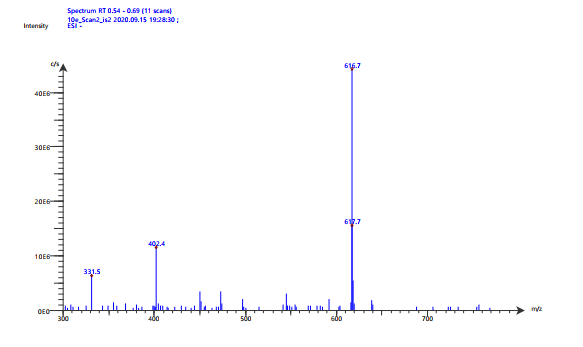


**H-NMR for compound 5f**


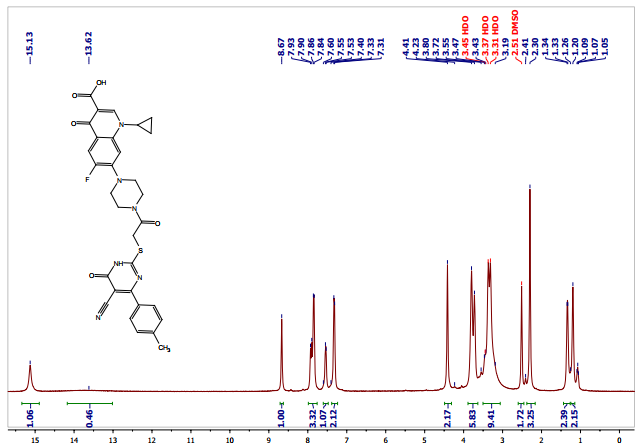


**13c-NMR for compound 5f**


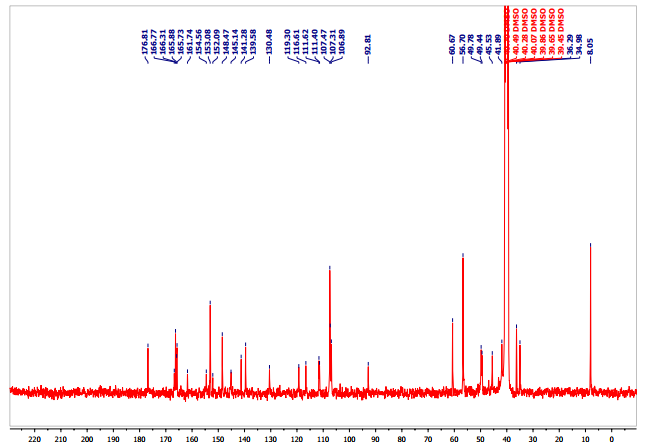


**Mass spectra of compound 5f**
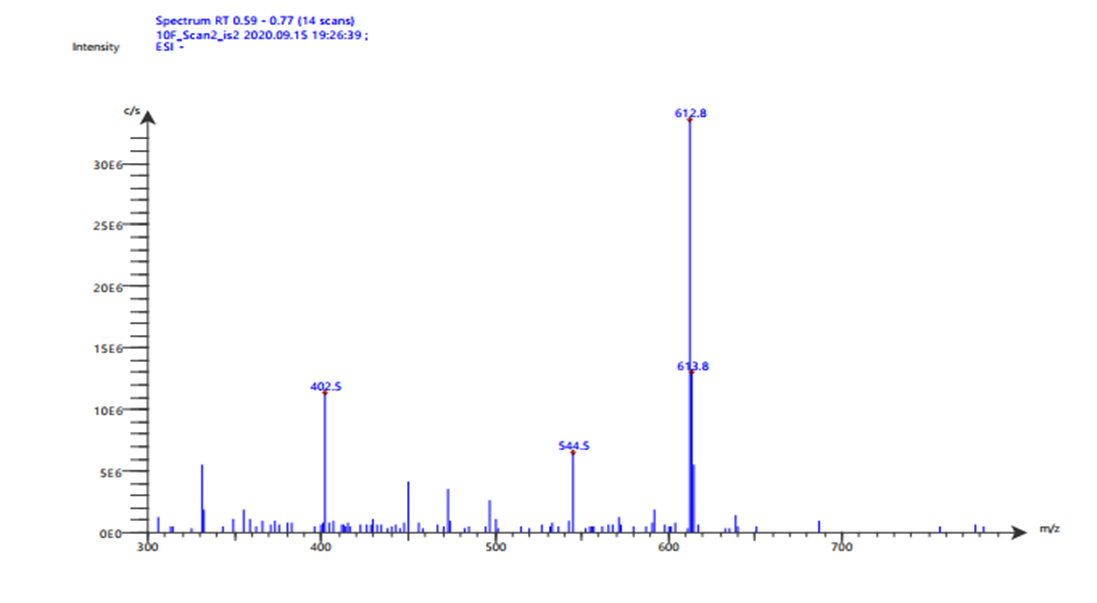


**H-NMR for compound 5g**


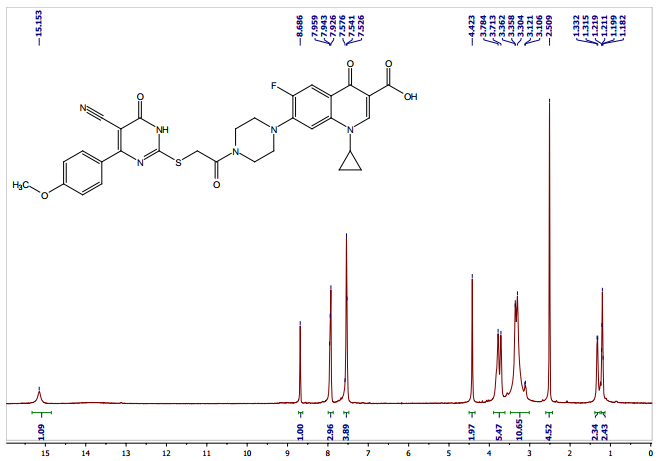


**13c-NMR for compound 5g**


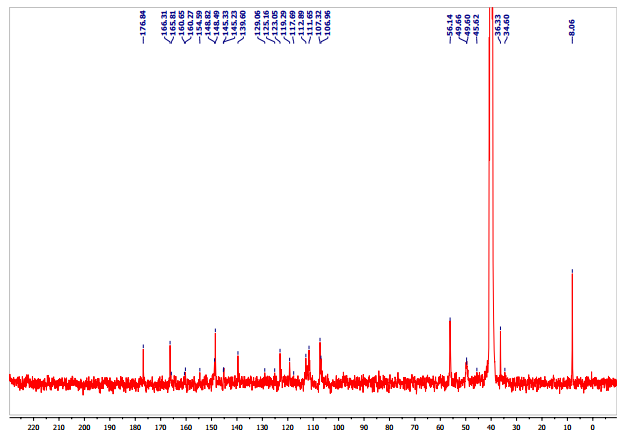


**Mass spectra of compound 5g**


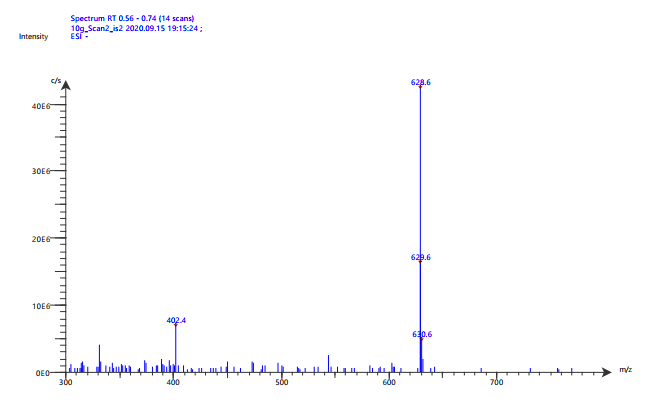


**H-NMR for compound 5H**


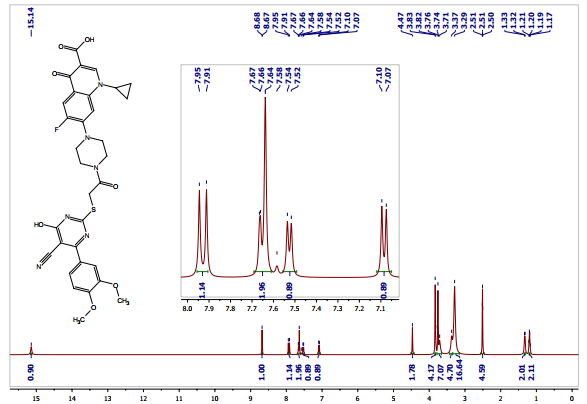


**13c-NMR for compound 5h**


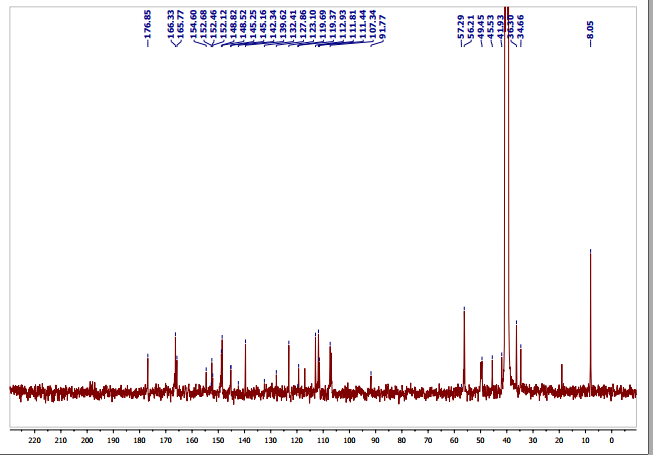


**Mass spectra of compound 5h**


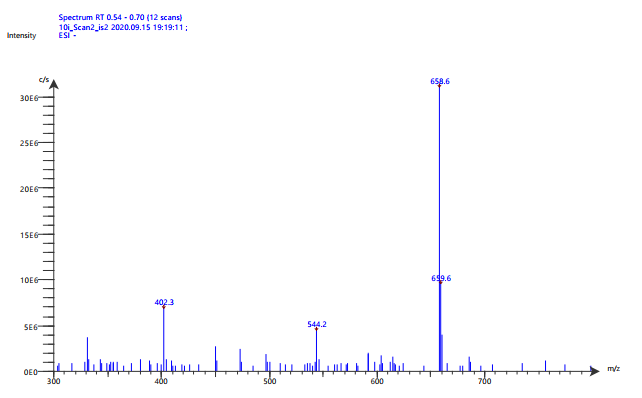


**H-NMR for compound 5i**


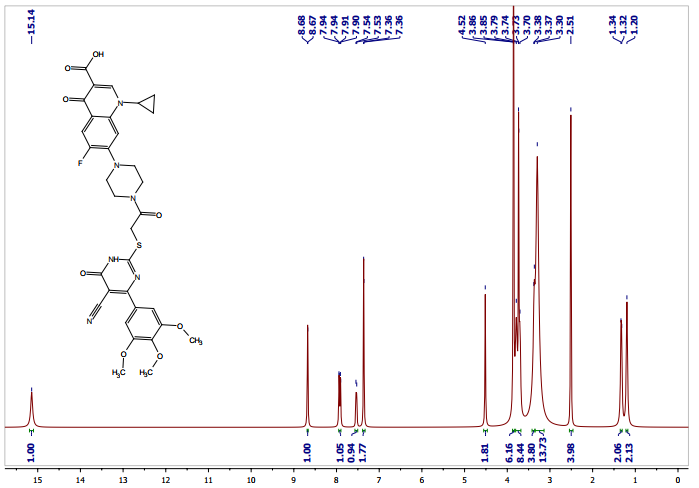


**13C-NMR for compound 5i**


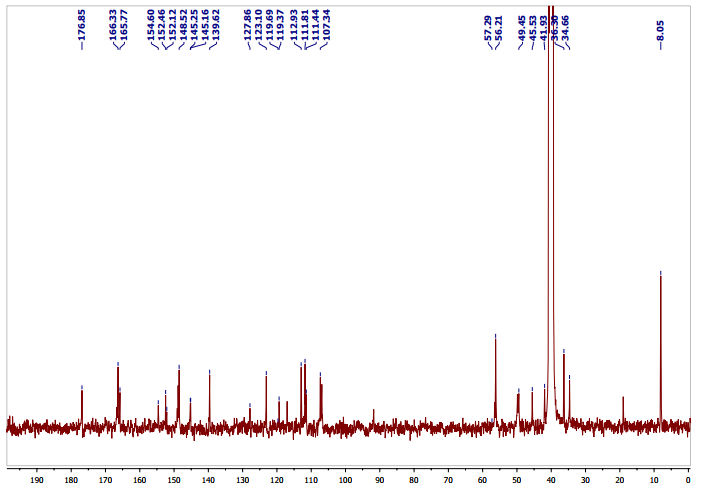


**Mass spectra of compound 5i**


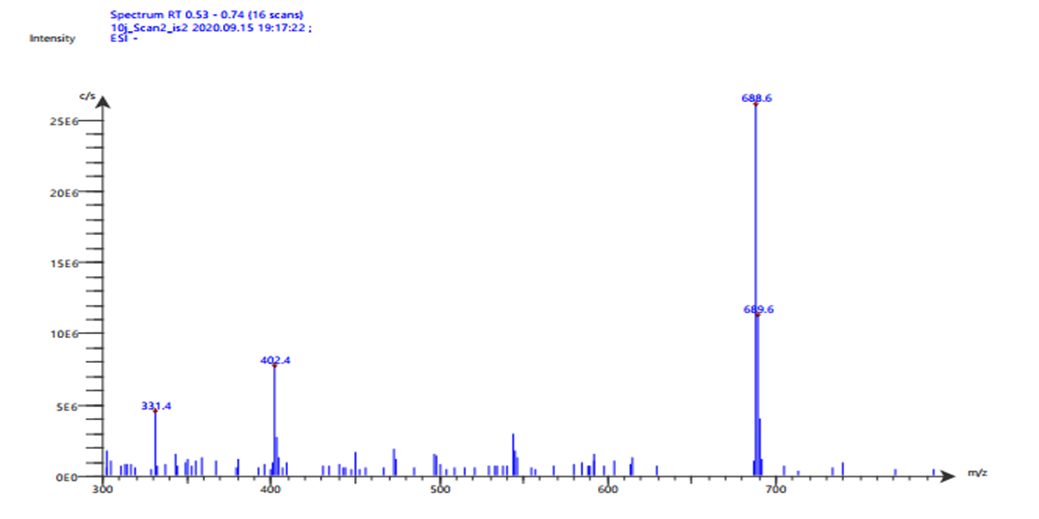


**Docking studies**


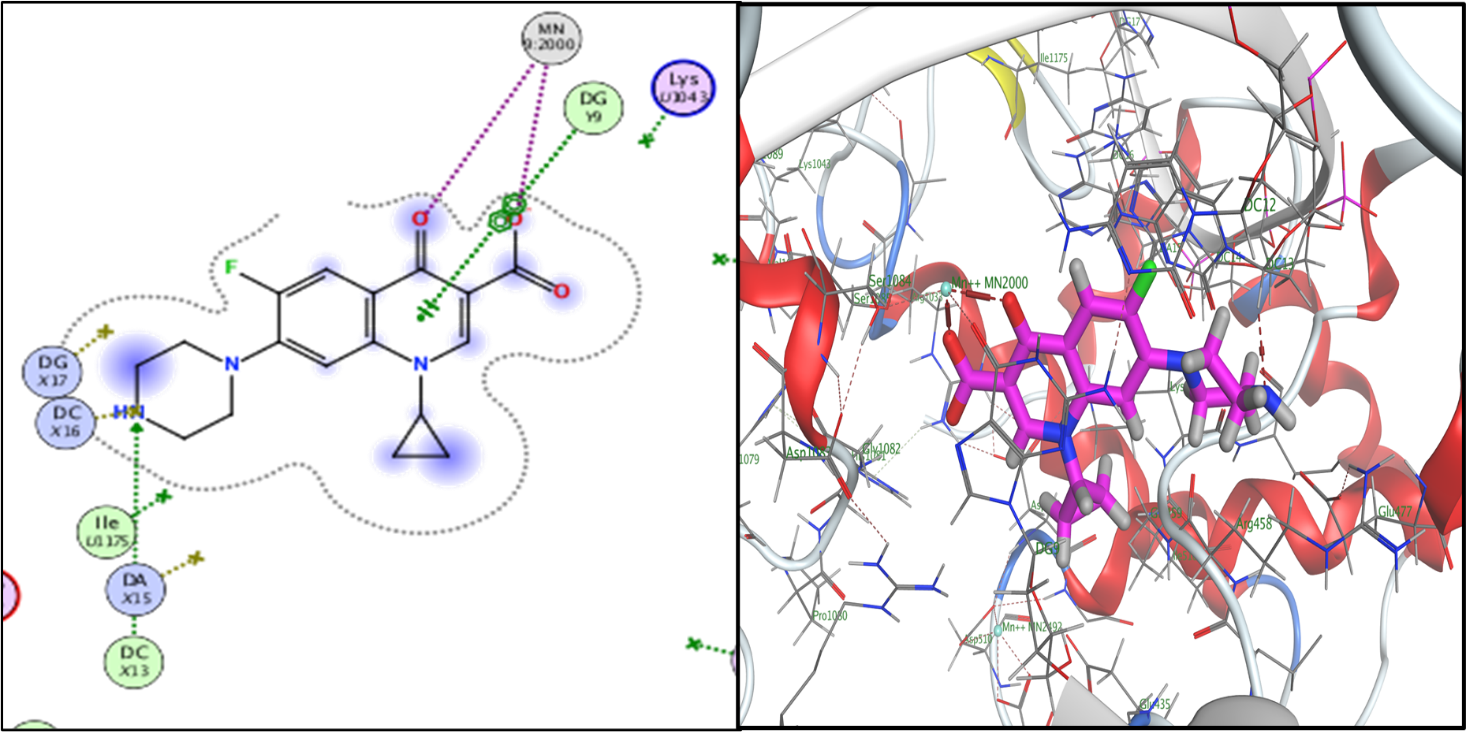


**Fig. 2.** 2D and 3Dbinding interactions of ciprofloxacin within gyrase active site (PDB:**2XCT)**‎

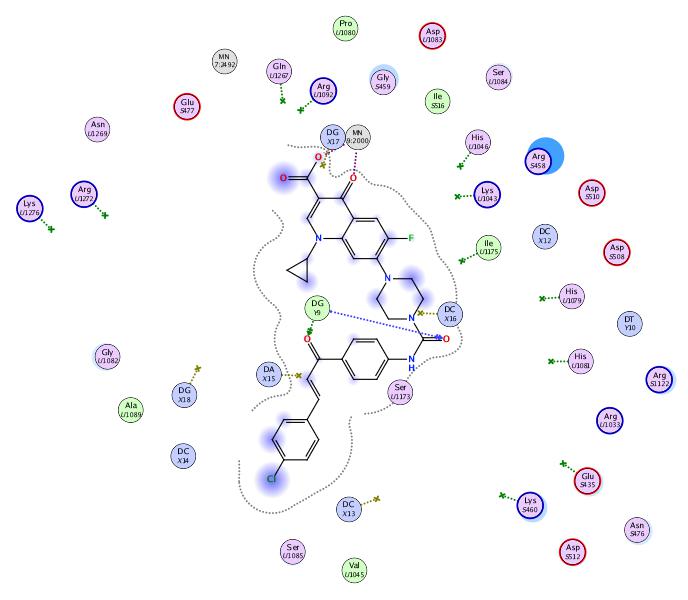
 **Fig.3.** 2D binding interactions of compound **2b** within gyrase active site (PDB:**2XCT**‎*).*

**Fig. 4** 2D binding interactions of compound **2c** within gyrase active site (PDB:**2XCT**‎*).*


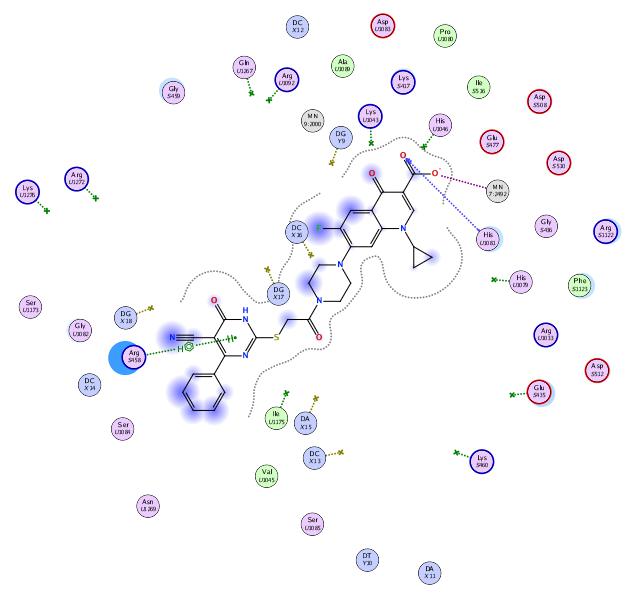


**Fig. 5** 2D binding interactions of compound **5a** within gyrase active site (PDB:**2XCT**‎*).*


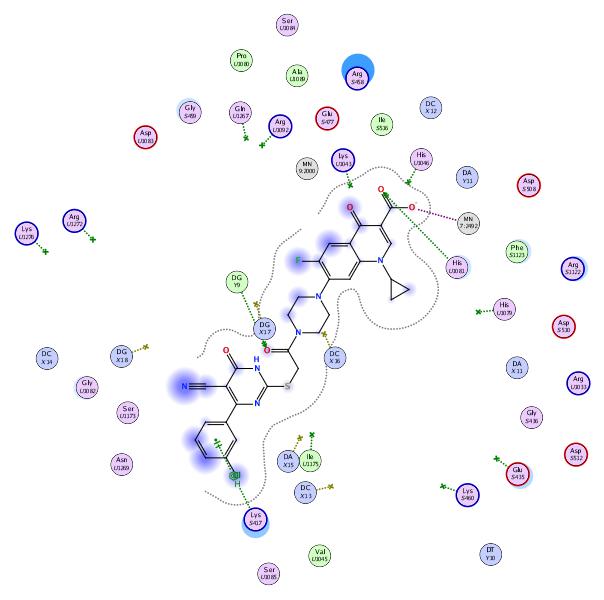


**Fig. 6** 2D binding interactions of compound **5b** within gyrase active site (PDB:**2XCT**‎*).*


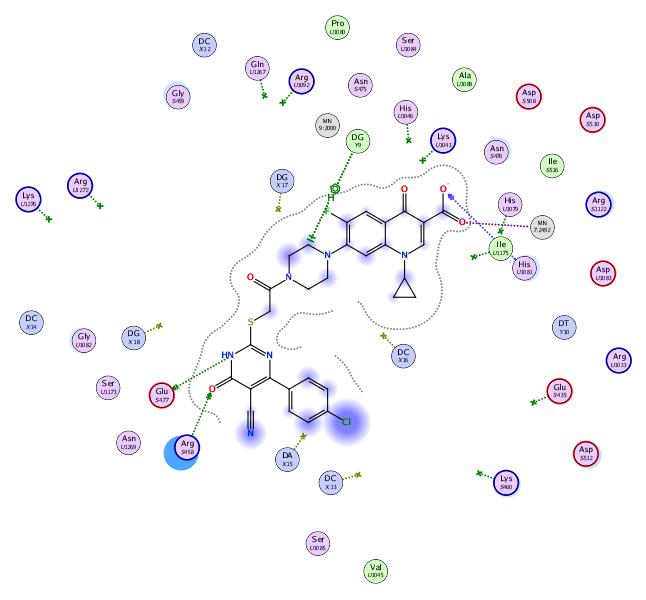


**Fig. 7** 2D binding interactions of compound **5c** within gyrase active site (PDB:**2XCT**‎*).*


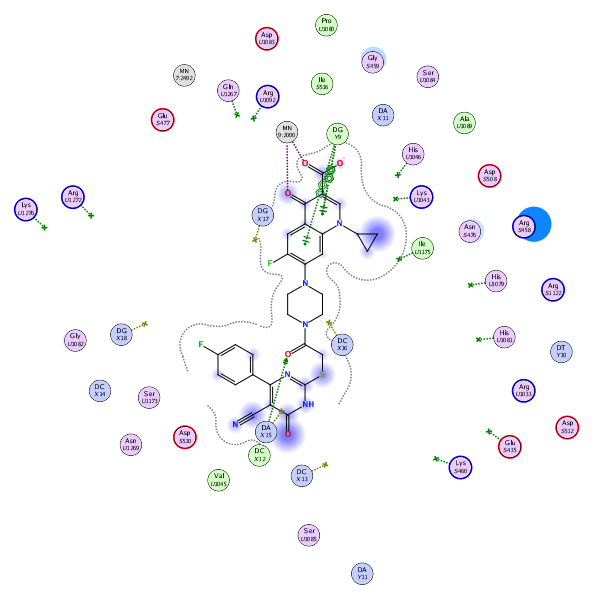


**Fig. 8** 2D binding interactions of compound **5e** within gyrase active site (PDB:**2XCT**‎*).*


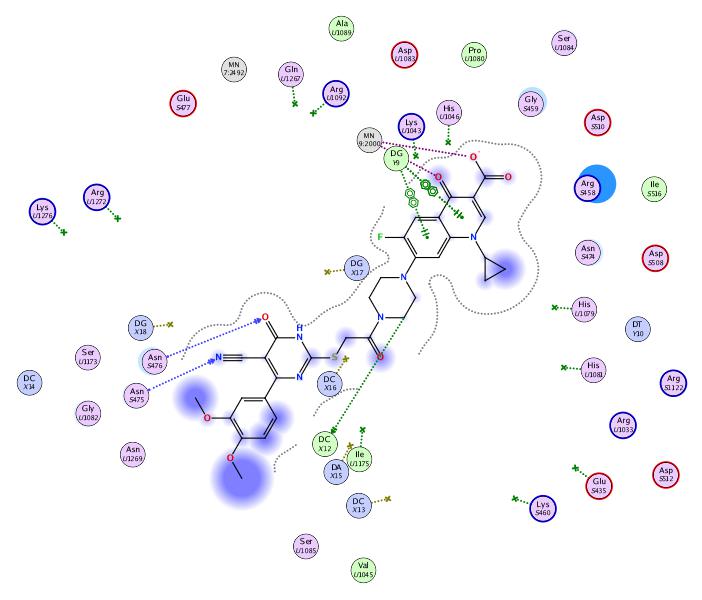


**Fig. 9** 2D binding interactions of compound **5h** within gyrase active site (PDB:**2XCT**‎*).*


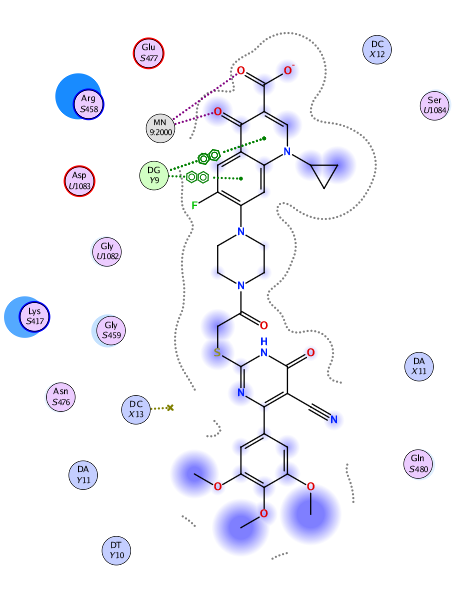


**Fig. 10** 2D binding interactions of compound **5i** within gyrase active site (PDB:**2XCT**‎*).*

**4.2. Biology**

**4.2.1. Screening of the antimicrobial activity.**

***4.2.2.1. Screening of the antibacterial activity.***

The antibacterial activity of compounds **1**, **2a-j**, and **5a-i** and ciprofloxacin were determined according to the standard agar cup diffusion method [1] at Deraya University, Faculty of Pharmacy, Department of Microbiology.

**4.2.2.1.1. Microbial strains and culture conditions**

Three bacterial species representing both Gram-positive and Gram-negative strains and were used to test the antibacterial activity of the newly synthesized ciprofloxacin derivatives. Standard strains of *Staphylococcus aureus* (ATCC 6538), *Pseudomonas aeruginosa* (ATCC 10145), *Escherichia coli* (ATCC 8739) were obtained from microbiological resource center, Faculty of Agriculture, Ain Shams University, Cairo, Egypt. All isolates were maintained at -70ºC in Trypticase Soya Broth (TSB, Becton, and Dickinson) with 10 % glycerol. Prior to inoculation, all isolates were subcultured at 37ºC for 24 h on Trypticase Soya Agar (TSA, Becton, and Dickinson) and TSB, respectively

**4.2.2.1.2. Determination of the minimum inhibitory concentration (MIC)**

From all the tested bacteria 0.5 mL of 1×108 CFU/mL (0.5 McFarland turbidity) were plated in sterile petri dishes, then 20 mL of Mueller Hinton Agar media (Oxoid) was added to each petri dish. The plates were rotated slowly to ensure uniform distribution of the microorganisms and then allowed to solidify on a flat surface. After solidification, four equidistant and circular wells of 10 mm diameter were carefully punched using a sterile cork bore. Two-fold serial dilutions of the tested compounds using DMSO were performed. An equal volume of 100µL of each dilution was applied separately to each well in three replicates using a micropipette. All plates were incubated at 37 ºC for 24 h. The inhibition zones were measured, and their average was calculated. The MIC was calculated by plotting the natural logarithm of the concentration of each dilution of the tested compounds against the square of zones of inhibition and a regression line was drawn through the points then the antilogarithm of the intercept on the logarithm of concentration axis gave the MIC value [2].

***4.2.2.2. Screening of antifungal activity.***

The antifungal activity of compounds **1**, **2a-j** and **5a-i** and ketoconazole were determined according to the agar cup diffusion method [1, 3] at Deraya University, Faculty of Pharmacy, Department of Microbiology.

**4.2.2.2.1. Fungal strains and culture conditions**

*Candida albicans* was used for screening of the antifungal activity of the newly synthesized ciprofloxacin derivatives. Standard strain of *Candida albicans* (ATCC 10231) was obtained from microbiological resource center, Faculty of Agriculture, Ain Shams University, Cairo, Egypt. The isolate was maintained at -70ºC in Trypticase Soya Broth (TSB, Becton, and Dickinson) with 10 % glycerol. Prior to inoculation, the isolate was subcultured at 37ºC for 24 h on Trypticase Soya Agar (TSA, Becton, and Dickinson) and TSB, respectively

**4.2.2.2.2. Determination of the minimum inhibitory concentration (MIC)**

From the tested *Candida albicans* 0.5 mL of 1×108 CFU/mL (0.5 McFarland turbidity) was plated in sterile petri dishes, then 20 mL of Sabouraud agar was added to each petri dish. The plates were rotated slowly to ensure uniform distribution of the microorganisms and then allowed to solidify on a flat surface. After solidification, four equidistant and circular wells of 10 mm diameter were carefully punched using a sterile cork bore. Two-fold serial dilutions of the tested compounds using DMSO were performed. An equal volume of 100µL of each dilution was applied separately to each well in three replicates using a micropipette. All plates were incubated at 37 ºC for 24 h. The inhibition zones were measured, and their average was calculated. The MIC was calculated by plotting the natural logarithm of the concentration of each dilution of the tested compounds against the square of zones of inhibition and a regression line was drawn through the points then the antilogarithm of the intercept on the logarithm of concentration axis gave the MIC value [1, 3].

***4.2.2.3. Staphylococcus aureus DNA Gyrase Supercoiling Assay***

*S. aureus* DNA gyrase assay was performed according to established protocols obtained from inspirals (Cat. No. SAS4001) The new compounds and ciprofloxacin were dissolved in DMSO and serially diluted at concentrations of 100, 10, 1 and 0.1μM, and then assayed in reaction mixtures in three different replicate runs.

*S. aureus* DNA gyrase was incubated at 37°C for 30 min in a total reaction volume of 30 μl containing 40 mM HEPES. KOH (pH 7.6), 10 mM magnesium acetate, 10 mM DTT, 2 mM ATP, 500 mM potassium glutamate, 0.05 mg/ml albumin and Relaxed pBR322. DNA gyrase supercoiling reactions catalyzed by *S. aureus* gyrase, Stop reaction by adding 30 μL of STEB and 30 μL of chloroform/isoamyl alcohol (v:v, 24:1),and then Vortex briefly ~5 secs and centrifuge for 1 min. after which 20 µL of this was loaded on a 1% agarose gel that was then run at ~75V for approximately 2 hours. The gel was stained by (0.5 mg/L) ethidium bromide in water. Fluorescent images were taken at a wavelength of 300 nm on a UV transilluminator imaging system. the fluorescence intensity of the supercoiled plasmid reaction product was quantitated using ImagQuant software (Molecular Dynamics). The results as IC50 values (concentration of the tested compound that leads to50% inhibition of enzyme activity) for all samples were determined by nonlinear regression analysis inGraphPad Prism.

**4.3. Docking Studies on bacterial gyrase enzymes**

All the compounds were drawn in Chem Draw professional (ver. 2015), converted to smiles, transferred to Molecular Operating Environment (MOE 2019) program. Hydrogens were added and finally the energy of the docked structures ‎was minimized using MMF94FX forcefield with a gradient RMS of 0.001kcal/mol. Bacterial gyrase in complex with DNA and ciprofloxacin (2XCT) protein was downloaded from the RCSB Protein Data Bank (https://www.rcsb.org/). Bacterial gyrase in complex with DNA and ciprofloxacin X-ray crystal structure with 3.35 Å resolution (PDB ID: 2XCT) was used for docking studies. The protein was prepared by using the MOE quickprep protocol. The ligands were ‎then docked in the binding‏ ‏site using the triangle matcher placement method. The number of generated poses was set for 10 for each ligand and default settings were employed for other parameters. Refinement was ‎carried out using Forcefield and‏ ‏scored using the affinity ΔG scoring system. To validate the docking study at the 2XCT active site, the co-crystallized ligand was re-docked into the binding site using the same set of parameters as described above. The resulting docking poses were visually inspected, and the poses of the lowest binding free energy value and with the best hydrophobic, H-bonding, and electrostatic interactions within the binding pocket of target protein.

**4.4. Determination of solubility and lipophilicity**

- - 1. ***UV Spectrophotometric Scanning of drug***

A sample of drug was accurately weighed and dissolved in 100 ml of distilled water to prepare 100 mcg/ml stock solutions. Appropriate dilution of the stock solution with phosphate buffer pH 6.8 and 7.8 was then made to prepare a working solution of 40mcg/ml. The absorbance of the drug in the working solution was scanned in the ultraviolet region (200-400 nm) to determine the wavelength of maximum absorbance (λ_max_= 272 nm).

- - 1. ***Construction of UV Calibration curve*:**

After determination of λ max of drug, a calibration curve was constructed by preparing solutions containing different concentrations of (1-5 mcg/ml) from stock solution after appropriate dilution with phosphate buffer pH 6.8. The UV absorbance of the prepared sample solutions were measured at the predetermined λ max using phosphate buffer pH 6.8 as a blank.

- - 1. ***Solubility determinations***

Aqueous solubility of the complexes was measured as a function of pH. An excess of each derivative was placed into suitable stoppered containers. Seven of these containers (in triplicate) were added with variable volumes of phosphate buffer to obtain pH values of 6.8 and 7.8. The samples were immersed in a water bath thermostatized at 37±1^◦^C and 100 rpm and periodically shaken for 48h. Once the equilibrium was reached, the pH of the supernatant was regarded. Aliquots of the filtrate properly diluted with suitable buffer were analyzed by UV spectrophotometry (Shimadzu *UV A-160)* at the maximum wavelengths (λ _max_ 272 nm). [59, 60]

***Determination of partition coefficients***

The n-octanol/water apparent partition coefficient at the isoelectric point (or distribution constant) was measured by using the traditional shake-flask technique. The pH of the aqueous buffer was first adjusted to isoelectric point of the compound. The n-octanol and aqueous phases were then mutually saturated before the measurement. The compound was dissolved in aqueous buffer solution and the solution was equilibrated with n-octanol for 1 h. The aqueous concentration to n-octanol concentration ratio (C_aq_/C_oct_) was 1:1. The samples were agitated for 5hrs at water bath shaker thermostatized at 25±1^◦^C and 100 rpm. The concentration of the solute was determined in the aqueous and organic phases at the isoelectric point by UV spectroscopy the log p value was calculated as follows:

$$\log p=\frac{[conc.aq]}{[conc. oct]}$$
